# Discovery of Late Mississippian (late Serpukhovian)–Early Pennsylvanian (earliest Bashkirian?) foraminiferal assemblages from the Sanandaj–Sirjan Zone, Iran: Biostratigraphic and palaeoenvironmental implications

**DOI:** 10.1002/gj.4641

**Published:** 2022-11-23

**Authors:** Shirin Fassihi, Elena Kulagina, Petra Heinz, Fariba Shirezadeh Esfahani

**Affiliations:** ^1^ Department of Palaeontology University of Vienna Vienna Austria; ^2^ Department of Earth Sciences Institute of Geology, Ufa Federal Research Centre of the Russian Academy of Sciences Ufa Russia; ^3^ Department of Earth Sciences Azad University of Tehran Tehran Iran

**Keywords:** foraminiferal faunas, Ghaleh Formation, Iran, Sanandaj–Sirjan zone, Shahreza region

## Abstract

This study reports the new discovery of relatively abundant foraminiferal faunas from the upper Serpukhovian–lowermost Bashkirian? of the Ghaleh Formation in the Shahreza region of the Sanandaj–Sirjan Zone, Iran. Four successive assemblages spanning the upper Serpukhovian–lowermost Bashkirian? are proposed: (1) Assemblage with *Biseriella minima* and *Eostaffellina paraprotvae*; (2) Assemblage with *Bradyina cribrostomata*; (3) Assemblage with *Parastaffella utkaensa* and *Plectostaffella* spp., (4) Assemblage with *Plectostaffella* ex gr. *varvariensis*. The newly discovered foraminiferal assemblages of the Sanandaj–Sirjan Zone have some species in common with assemblages of the Russian Platform, Donets Basin, Urals, and Western Europe. *Ikensieformis* aff. *mirifica*, and *Eostaffella igoi*, and a new species *Ikensieformis persiaensis* sp. nov. are described. The microfacies analysis of the Ghaleh Formation limestones suggests a moderate to high‐energy shallow marine warm environment, more likely of the inner ramp.

## Graphical abstract

The new discovery of the Late Mississippian (late Serpukhovian)–Early Pennsylvanian (early Bashkirian?) foraminiferal assemblages, providing the palaeoecological data about the upper Serpukhovian–lowermost Bashkirian? foraminiferal faunas, and identifying the depositional environments of the Sanandaj–Sirjan Zone during this time, as well as describing the new species *Ikensieformis persiaensis* sp. nov. are among the main outcomes obtained in this study.

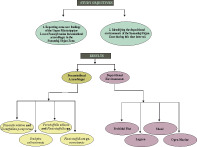

## 1 INTRODUCTION

Carboniferous foraminifers were first reported in Iran by Bozorgnia (1973) from the Alborz Mountains of Northern Iran. They were mostly characterized by the Tournaisian–Viséan smaller foraminifers, however, a few Moscovian fusulinoids were also described from the eastern part of the Alborz Mountains, without any clearly defined descriptions (Ueno, 2022). Further Carboniferous fusulinoids were also reported by Lys et al. (1978) from the Alborz Mountains. However, as stated subsequently by Vachard (1996), the Carboniferous fusulinoids, except for the Mississippian ones in the Alborz, were not considered to be a substantial issue in the Carboniferous study of Iran. This century, however, several sections suitable for the analysis of Mississippian and Pennsylvanian fusulinoids have been examined in different regions of Iran (including Central and East Iran, and the Sanandaj–Sirjan Zone). The Carboniferous fusulinoids were studied in detail by Iranian geologists and foreign palaeontologists in Central and East Iran (e.g., Leven et al., 2006; Leven & Gorgij, 2006; Leven & Taheri, 2003).

Carboniferous marine deposits are widely distributed in the Sanandaj–Sirjan Zone, where the studied biostratigraphic section is located. However, except for a few studies (e.g., Baghbani, 1993; Fassihi, 2017; Fassihi et al., 2017, 2018, 2019; Fassihi & Shirezadeh, 2018; Leven & Gorgij, 2008b), the foraminiferal faunas have not been investigated in detail. The age of the Carboniferous interval in this area is mainly determined based on conodonts (Bahrami et al., 2014; Boncheva et al., 2007). Recently, the age of the Lower–Upper Carboniferous sequence in this tectonic unit has also been substantiated, based on the foraminifera (Fassihi et al., 2014, 2017, 2018, 2020; Fassihi & Shirezadeh, 2018; Leven & Gorgij, 2008a).

The presence of Serpukhovian foraminifers in the Sanandaj–Sirjan Zone was first described in preliminary reports by Fassihi et al. (2017) and Fassihi and Shirezadeh (2018). The current study exhaustively delineates the Serpukhovian foraminiferal faunas in this tectonic block.

As indicated by Ueno (2022), the Iranian Carboniferous sequence is currently a major target for the study of fusulinoids. Therefore, an interpretation of the Upper Serpukhovian–lowermost Bashkirian? fusulinoids in the Sanandaj–Sirjan Zone are not only valuable for biostratigraphic correlation with the well‐known type sections of the same age in the Russian Platform, Donets Basin, Urals, Western Europe, and North America, but also for reconstructing the depositional environments of the Sanandaj–Sirjan Zone during this time interval.

The purposes of this paper are (1) to report some new findings of the Late Mississippian (late Serpukhovian)–Early Pennsylvanian (earliest Bashkirian?) foraminiferal assemblages in the Sanandaj–Sirjan Zone, (2) to provide palaeoecological data about the upper Serpukhovian–lowermost Bashkirian? foraminiferal faunas in this area; and (3) to identify the depositional environments of the Sanandaj–Sirjan Zone in this time interval.

## 2 GEOLOGICAL SETTING AND STRATIGRAPHY

**FIGURE 1:**
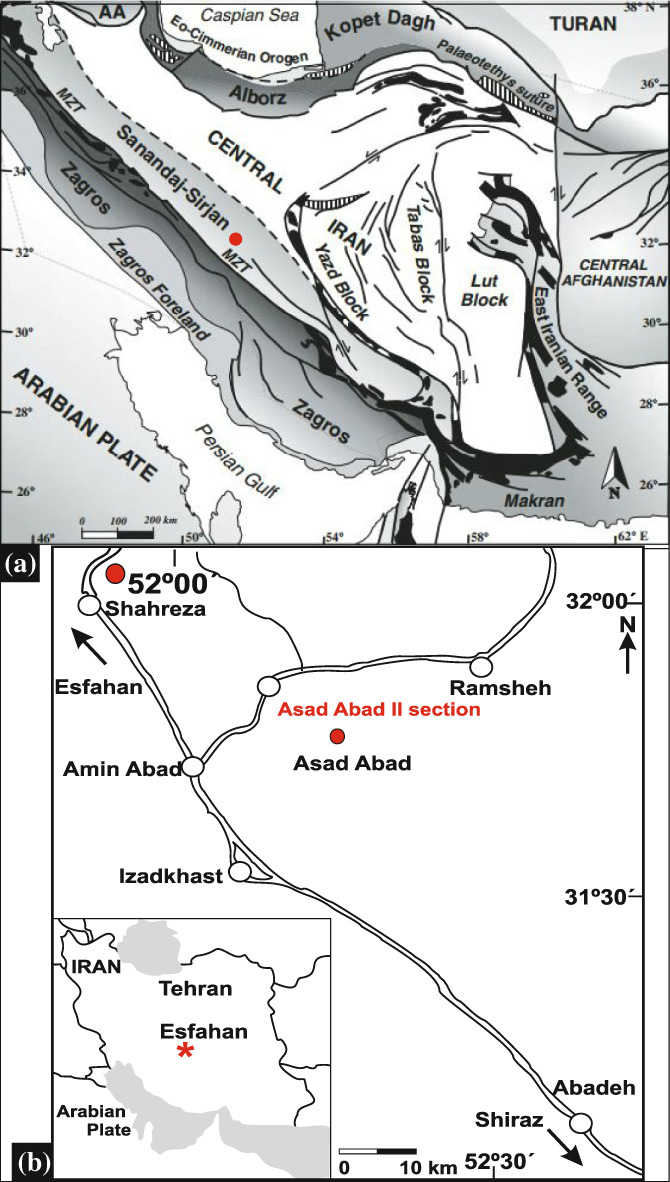
(a) Tectonic subdivisions of Iran showing the location of the Asad Abad II section (base map after Angiolini et al., 2007); (b) map of the Shahreza area showing the location of the Asad Abad II section (modified after Hampe et al., 2013)

Iran, with its complicated geological structure, is divided into several tectonic blocks, including the Sanandaj–Sirjan Zone, Alborz, Zagros, East Iran (Lut and Tabas blocks), and Central Iran (Yazd Block) (Arfania & Shahriari, 2009; Ruban, 2007; Torsvik & Cocks, 2004) (Figure 1a).

The Sanandaj–Sirjan Zone is principally formed of metamorphic complexes and granitoid intrusions. It comprises Carboniferous–Permian strata that show various lithological features compared to the tectonic blocks of East and Central Iran (Ahadnejad, 2013; Alavi & Kishvar, 1991; Bahrami et al., 2014). The foraminiferal study in this work is based on samples collected from one stratigraphic section, that is, the Asad Abad II section. This section structurally belongs to the Carboniferous and Permian strata of the Shahreza‐Hambast‐Abadeh Belt (Leven & Gorgij, 2008a) and is situated approximately 35 km southeast of the town of Shahreza (Figure 1b). Its starting point is at the coordinates: N31°46′13.4″; E52°08′56.9″.

In accordance with Leven and Gorgij (2011b), the lithostratigraphic subdivisions of the Carboniferous strata in the Sanandaj–Sirjan Zone are as follows.

The Mississippian strata are represented by sequences of limestone which are intercalated with shale and sandstone in the upper part. The Tournaisian, Viséan, and Serpukhovian strata correspond to the Shishtu Group of the Tabas Block sections in East Iran. In the report of Leven and Gorgij (2011b), the Shishtu Group consists of the Shishtu 1 and Shishtu 2 formations. The Frasnian–early Tournaisian? Shishtu 1 Formation is composed of sandstones and shale intercalated with limestones. Shishtu 2 Formation is mostly composed of carbonate strata. Stöcklin (1971) dated this formation as Tournaisian–early Viséan. However, according to Leven and Gorgij (2011b), the age of the Shishtu 2 Formation was revised as late Tournaisian–Serpukhovian. The Pennsylvanian sequence in the Sanandaj–Sirjan Zone is defined by Leven and Gorgij (2011b) as the Sardar Group. As Leven and Gorgij (2011b) declared, the Sardar Group unconformably overlies the Shishtu Group and consists of the Ghaleh and Absheni formations, which correspond roughly to the Bashkirian and Moscovian stages. However, the conodont data (Boncheva et al., 2007) and the foraminiferal evidence (Fassihi et al., 2017, 2018; this study) show that the stratigraphic range of the Ghaleh Formation is from the upper Serpukhovian–lowermost Moscovian, rather than the Bashkirian. The Ghaleh Formation is composed chiefly of limestone, with argillite, aleurolite, and sandstone intercalations. Basal conglomerates of this formation rest on the sandy limestone of the Shishtu Group.

**FIGURE 2:**
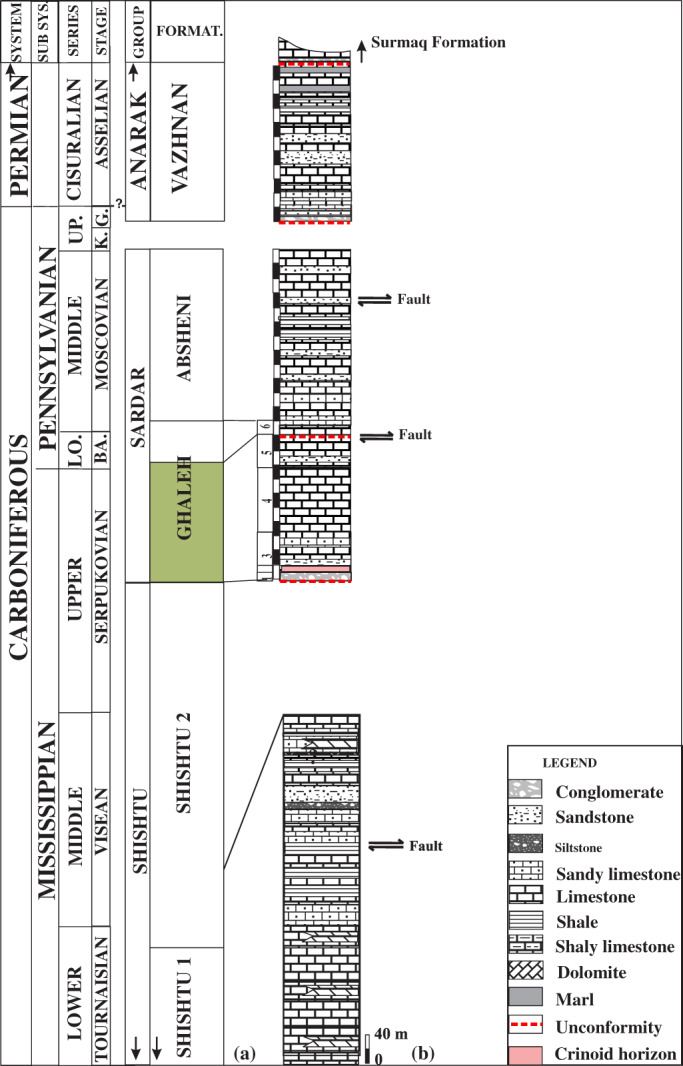
(a) The lithostratigraphic subdivisions of the Carboniferous–Lower Permian strata at the Asad Abad II section (modified after Fassihi, 2017, note that the Shishtu group is adapted from Boncheva et al., 2007); (b) the stratigraphic column of the Mississippian–Lower Permian strata at the Asad Abad II section. BA, Bashkirian; G, Gzhelian; K, Kasimovian; LO, lower; UP, upper. The interval in question in this study is shown by the green colour.

The Absheni Formation of the lower–upper Moscovian age is dominated by limestone in the lower part and by shale in the upper part. Abundant fusulinoids in this formation are characteristic of the Kashirian and Podolskian substages of the Moscovian Stage (Leven & Gorgij, 2011b). This is overlain by a conglomerate bed of the Anarak Group. This conglomerate bed was considered by Baghbani (1993) as the basal part of the Vazhnan Formation (now known to be of Gzhelian–Asselian age, see Leven & Gorgij, 2011a) (Figure 2a).

## 3 RESULTS

### 3.1 Lithostratigraphy

Note that the Asad Abad II section is not the same as the original Asadabad section of Boncheva et al. (2007), but was newly introduced by Fassihi et al. (2017). Owing to the very complicated nature of this area which is the result of the tectonic movements of the Shahreza region, it is very difficult to correlate the original Asadabad section of Boncheva et al. (2007) with the Asad‐Abad II section.

The Ghaleh Formation (upper Serpukhovian–lowermost Moscovian) with a thickness of 217 m is divided into six informal units (Figure 2b). This study is focused on units 1–5 of the succession, which is late Serpukhovian–earliest Bashkirian? in age, with a thickness of about 203 m. Fusulinoids of Unit 6, of latest Bashkirian−earliest Moscovian age, were already described by Fassihi et al. (2017).

The studied interval begins with the basal polymictic conglomerate. It contains sandstone and a small proportion of shaly limestone, but most of the formation is composed of bioclastic limestones intercalated with sandstone. These strata lie unconformably over an erosional surface, capping the underlying beds of the lower Viséan Shishtu 2 Formation. The top of the interval at issue is unconformably overlain by sediments of the same formation (Unit 6) of the Bashkirian–lowermost Moscovian (Fassihi et al., 2017). The results of the study of the middle–late Bashkirian foraminifers are currently being prepared for publication by Fassihi et al.

### 3.2 Biostratigraphy of the Asad Abad II section

The identified foraminifers in this study mainly occur in units 3–5. These strata, together with about 11 m of the crionoid horizon and 13 m conglomerate, cap the strata of the lower Viséan beds with a stratigraphic unconformity corresponding to the middle Viséan–early Serpukhovian (see Figure 2). The Visean deposits of the Asad Abad II section were originally assigned to two local biozones: (1) *Uralodiscus rotundus–Glomodiscus miloni* Zone corresponding to the MFZ11B and (2) *Lapparentidiscus bokanensis* Zone corresponding to the MFZ12 foraminiferal biozone of the stratotype area of the Viséan Stage of the Dinant Basin in Belgium (Fassihi et al., 2018; Fassihi & Shirezadeh, 2018). However, as declared by Cózar et al. (2020) in a revision of the lower–middle Viséan boundary interval of the key sections of the Paleotethys, the *L*. *bokanensis* Zone should be assigned to the lower Viséan Cf4γ‐δ subzones. Accordingly, owing to the absence of the index foraminifers of a middle Viséan age within the Mississippian assemblage in the Sanandaj–Sirjan Zone, the Cózar et al. (2020) opinion is followed in this paper.

By lateral correlation of Unit 1 (the basal conglomerate) and Unit 2 (the crinoid horizon) of the Asad Abad II section with the similar units reported by Boncheva et al. (2007) and Bahrami et al. (2014), in the Asadabad and Tang‐e‐Darchaleh sections respectively (both sections are located in the Sanandaj–Sirjan Zone), units 1 and 2 are tentatively assigned to the late Serpukhovian.

This interval contains interesting and rather well‐preserved assemblages of encrusting forms of smaller foraminifers and fusulinoids. Most of the discovered foraminiferal species are characteristic of the late Viséan–Serpukhovian. The species characteristic of the late Serpukhovian and Bashkirian are also present. In total, the assemblage contains 61 species of 28 genera, mainly belonging to eight foraminiferal orders, that is, Ammodiscida, Parathuramminida, Earlandiida, Archaediscida, Endothyrida, Palaeotextulariida, Ozawainellida and Staffellida. The identified foraminifers in the Asad‐Abad II section suggest that the host deposits correspond to the upper Serpukhovian and probably the lower Bashkirian.

**FIGURE 3:**
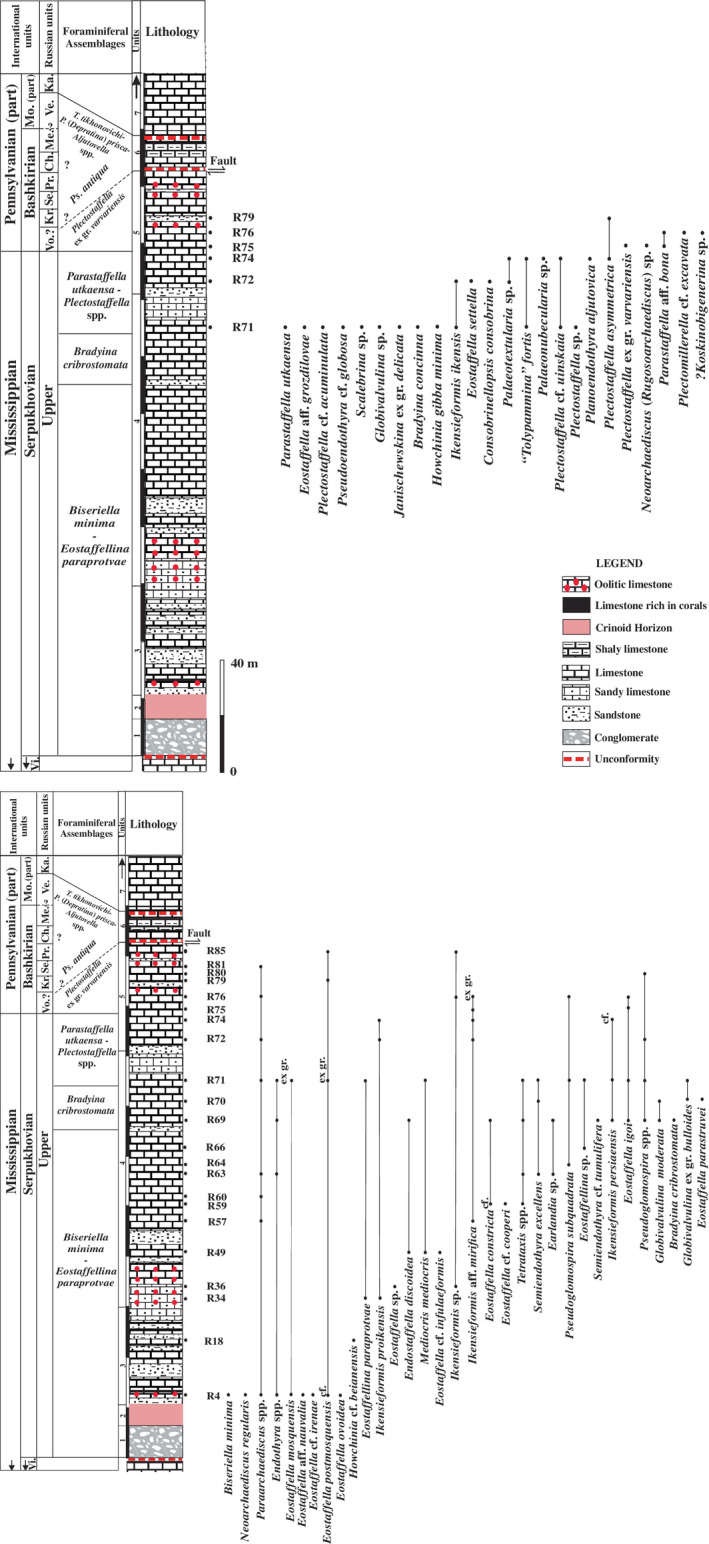
Stratigraphic occurrences of the late Serpukhovian–earliest Bashkirian? Foraminiferal faunas in the Asad Abad II section. Ch, Cheremshanian; Ka, Kashirian; Kr, Krasnopolyanian; Me, Melekessian; Mo, Moscovian; Pr, Prikamian; *Ps*. *antiqua*, *Pseudostaffella antiqua*; Se, Severokeltmian; *T*. *tikhonovichi‐P*. (*Depratina*) *prisca‐Aljutovella* spp. Zone, *Tikhonovichiella tikhonovichi‐Profusulinella* (*Depratina*) *prisca*‐*Aljutovella* spp. Zone; Ve, Vereian; Vi, lower Viséan; Vo, Voznesenkian

**FIGURE 4:**
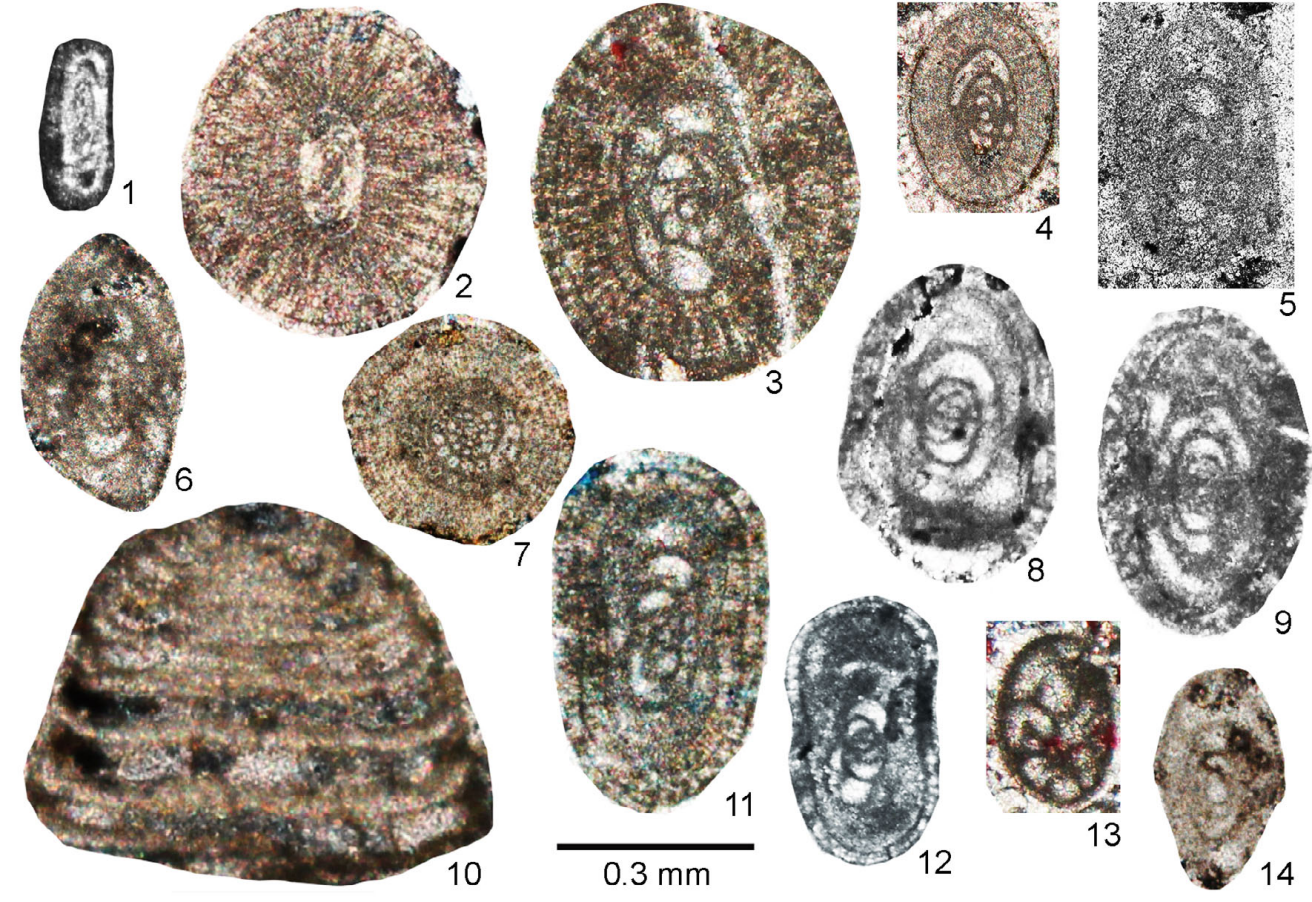
Selected late Serpukhovian–earliest Bashkirian? Foraminifers belonging to orders Endothyrida Fursenko, 1958; Archaediscida Poyarkov & Skvortsov, 1979 emend. Hance et al., 2011, and Ozawainellida Solovieva, 1980 from the Asad Abad II section. (1) *N*. *regularis* (Suleimanov, 1948), nearly axial section, SFA, no. 004/005, Spl. R4; (2) *Paraarchaediscus* sp. axial section, SFA, no. 004/006, Spl. R4; (3). *Endothyra* sp., oblique section, SFA, no. 004/007, Spl. R4; (4) *Eostaffella ovoidea* Rauser‐Chernousova, 1948a, axial section, SFA, no. 004/0012, Spl. R4; (5) *Eostaffellina paraprotvae* Rauser‐Chernousova, 1948b, axial section, SFA, no. 0034/001, Spl. R34; (6) *Ikensieformis proikensis* (Rauser‐Chernousova, 1948b), axial section, SFA, no. 0034/002, Spl. R34; (7) *Eostaffella* sp. median section, SFA, no. 0036/001, Spl. R36; (8) *Eostaffellina* cf. *irenae* Ganelina, 1956, oblique section, SFA, no. 004/0011, Spl. R4; (9) *Eostaffella mosquensis* Vissarionova, 1948, axial section, SFA, no. 004/008, Spl. R4; (10); *Howchinia* cf. *beianensis* Shen & Wang, 2016, nearly axial section, SFA, no. 0018/001, Spl. R18; (11) *Eostaffella* cf. *postmosquensis* Kireeva *in* Rauser‐Chernousova et al. (1951), slightly oblique section, SFA, no. 004/0013, Spl. R4; (12) *Eostaffella* aff. *Nauvalia* Rumjanzeva, 1970 SFA, no. 004/0015, Spl. R4; (13) *Biseriella minima* Reitlinger, 1950, median section, SFA, no. 004/009, Spl. R4; (14) *Ikensieformis* sp., axial section, SFA, no. 0036/003, Spl. R36.

**FIGURE 5:**
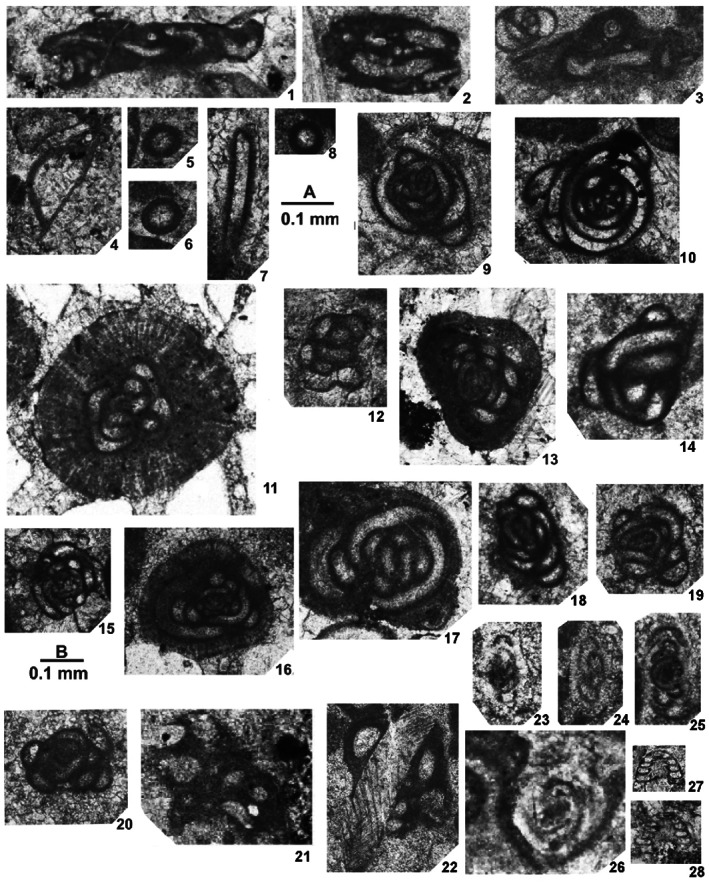
Selected late Serpukhovian–earliest Bashkirian? Foraminifers belonging to orders Ammodiscida Fursenko, 1958; Parathuramminida Mikhalevich, 1980; Earlandiida Cummings, 1955; and Archaediscida Poyarkov & Skvortsov, 1979 emend. Hance et al., 2011, From the Asad Abad II section. Figures 4─8, 23–28: Scale bar A; other figs: Scale bar B; Spl. = sample number. (1, 2, 3) ‘*Tolypammina*’ *fortis* Reitlinger, 1950, (1) SFA, no. 0074/004, Spl. R74, (2) SFA, no. 0071/009, Spl. R71, (3) SFA, no. 0074/005, Spl. R74; (4) *Tuberitina* sp., SFA, no. 0072/008, Spl. R72; (5)? *Pachysphaerina* sp., SFA, no. 0059/005, Spl. R59; (6, 7) *Earlandia* sp., (6) transverse section, SFA, no. 0063/001, Spl. R63, (7) longitudinal section, SFA, no. 0069/003, Spl. R69; (8) *Archaesphaera* sp. SFA, no. 0071/0036, Spl. R71; (9, 10) *Pseudoglomospira* aff. *postserenae* Brazhnikova *in* Aizenverg et al. (1983), (9) SFA, no. 0076/0010, Spl. R76, (10) SFA, no. 0072/0012, Spl. R72; (11, 19, 20) *Pseudoglomospira subquadrata* (Potievskaya & Vakarchuk *in* Brazhnikova et al., 1967), (11) SFA, no. 0064/001, Spl. R64, (19) SFA, no. 0076/0011, Spl. R76, (20) SFA, no. 0071/0010, Spl. R71; (12–17) *Pseudoglomospira* spp. (12) SFA, no. 0072/0013, Spl. R72, (13) SFA, no. 0072/009, Spl. R72, (14) SFA, no. 0071/0012, Spl. R71, (15) SFA, no. 0069/004, Spl. R69, (16) SFA, no. 0080/001, Spl. R80, (17) SFA, no. 0074/006, Spl. R74; (18) ‘*Pseudoglomospira*’ *multivoluta* Hance et al., 2011, SFA, no. 0069/005, Spl. R69; (21) *Palaeonubecularia* sp., subaxial section, SFA, no. 0074/007, Spl. R74; (22) *Scalebrina* sp., SFA, no. 0071/0037, Spl. R71; (23, 24) *Paraarchaediscus* sp., (23) subaxial section, SFA, no. 0071/0012, Spl. R71, (24) subaxial section, SFA, no. 0072/0010, Spl. R72; (25) *Paraarchaediscus vischerensis* (Grozdilova & Lebedeva, 1954), subaxial section, SFA, no. 0076/0012, Spl. R76; (26) *Neoarchaediscus* (*Rugosoarchaediscus*) sp., subaxial section, SFA, no. 0075/005, Spl. R75; (27, 28) *Howchinia gibba minima* Vdovenko, 1960, (27) axial section, SFA, no. 0071/0013, Spl. R71, (28) axial section, SFA, no. 0071/0014, Spl. R71.

**FIGURE 6:**
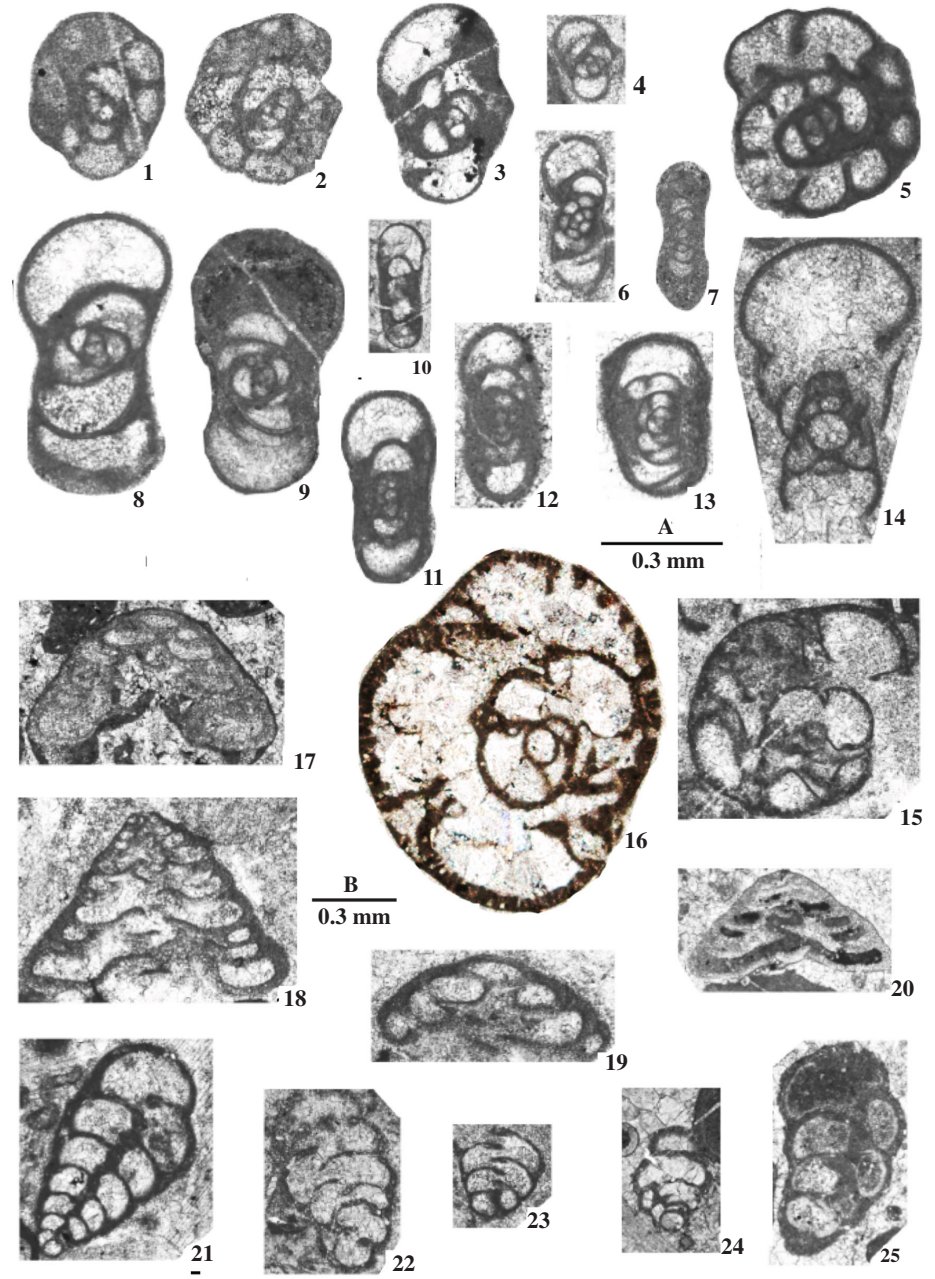
Selected late Serpukhovian–earliest Bashkirian? Foraminifers belonging to orders Endothyrida Fursenko, 1958; and Palaeotextulariida Hohenegger & Piller, 1975, from the Asad Abad II section. Figures 1–9: Scale bar A; other figures: Scale bar B; Spl. = sample number. (1, 2) *Endothyra* ex gr. *similis* Rauser‐Chernousova & Reitlinger *in* Rauser‐Chernousova et al. (1936), median sections, (1) specimen SFA, no. 0063/002, Spl. R63, (2) specimen SFA, no. 0069/006, Spl. R69; (3) *Semiendothyra* cf. *tumulifera* (Reitlinger, 1980) axial section, specimen SFA, no. 0069/007, Spl. R69; (4) *Endothyra paraprisca* Schlykova, 1951, subaxial section, specimen SFA, no. 0071/0015, Spl. R71; (5, 8, 9) *Semiendothyra excellens* (Zeller, 1953), (5) median section, specimen SFA, no. 0063/003, Spl. R63, (8) axial section, specimen SFA, no. 0070/001, Spl. R70, (9) axial sections, specimen SFA, no. 0071/0038, Spl. R71; (6) *Planoendothyra aljutovica* (Reitlinger, 1950), axial section, specimen SFA, no. 0074/008, Spl. R74; (7, 13) *Endostaffella discoidea* (Girty, 1915), (7) axial section, specimen SFA, no. 0049/001, Spl. R49, (13) oblique median section, specimen SFA, no. 0069/0012, Spl. R69; (10) *Mediocris breviscula* (Ganelina, 1951), axial section, specimen SFA, no. 0071/0016, Spl. R71; (11, 12) *Mediocris mediocris* (Vissarionova, 1948), (11) axial section, specimen SFA, no. 0071/0019, Spl. R71, (12) axial section, specimen SFA, no. 0049/002, Spl. R49; (14) *Janischewskina* ex gr. *delicata* (Malakhova, 1956), axial section, specimen SFA, no. 0071/0018, Spl. R71; (15) *Bradyina concinna* Reitlinger, 1950, median section, specimen SFA, no. 0071/0020, Spl. R71; (16) *Bradyina cribrostomata* Rauser‐Chernousova & Reitlinger *in* Rauser‐Chernousova and Fursenko (1937), slightly oblique section; specimen SFA, no. 0069/0012, Spl. R69; (17, 19, 20) *Tetrataxis* sp., (17) axial section, specimen SFA, no. 0059/001, Spl. R59, (19) axial section, specimen SFA, no. 0071/0017, Spl. R71, (20) axial section, specimen SFA, no. 0069/008, Spl. R69; (18) *Tetrataxis conica* Ehrenberg, 1854, emend. Nestler, 1973, near‐axial section, specimen SFA, no. 0063/005, Spl. R63; (21, 24, 25) *Palaeotextularia* sp., (21) axial section, specimen SFA, no. 0072/002, Spl. R72, (24) axial section, specimen SFA, no. 0072/003, Spl. R72; (25) axial section, specimen SFA, no. 0074/009, Spl. R74, (22)? *Koskinobigenerina* sp., axial section, specimen SFA, no. 0076/001, Spl. R76; (23) *Consobrinellopsis consobrina* (Lipina, 1948), axial section, specimen SFA, no. 0072/001, Spl. R72.

**FIGURE 7:**
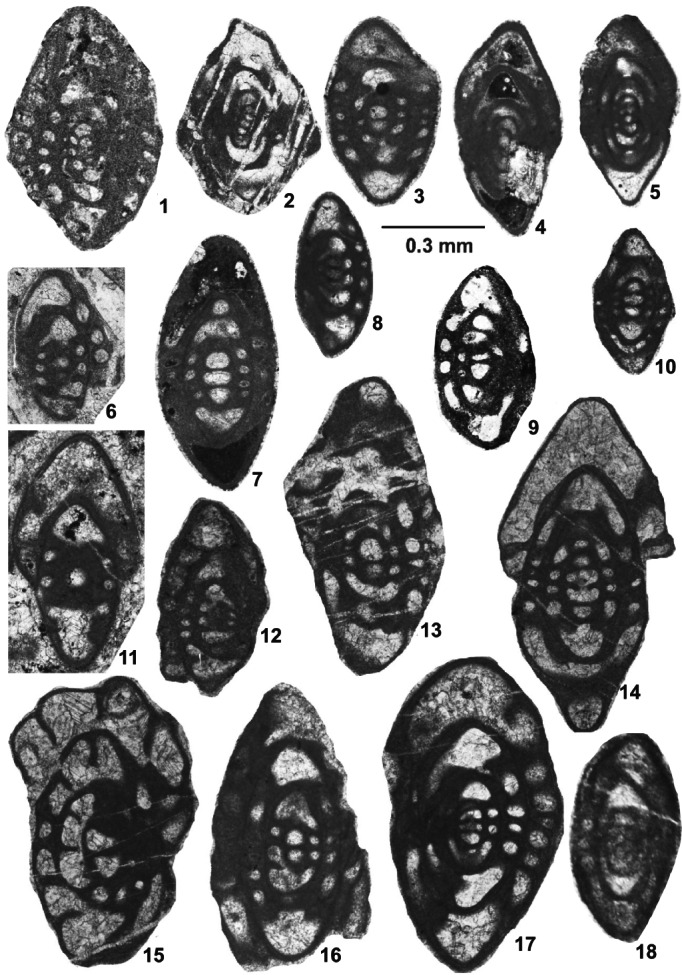
Species of *Ikensieformis* from the late Serpukhovian–earliest Bashkirian? Of the Asad Abad II section. Spl. = sample number. (1, 2, 3) *Ikensieformis ikensis* (Vissarionova, 1948), (1) axial slightly oblique section, specimen SFA, no. 0071/0023, Spl. R71, (2) tangential section, specimen SFA, no. 0072/004, Spl. R72, (3) axial section, specimen SFA, no. 0071/0024, Spl. R71; (4, 5, 10) *Ikensieformis proikensis* (Rauser‐Chernousova, 1948b), (4) axial section, specimen SFA, no. 0072/005, Spl. R72, (5) axial section, specimen SFA, no. 0072/0011, Spl. R72, (10) tangential section, specimen SFA, no. 0074/002, Spl. R74; (6) *Ikensieformis* ex gr. *mirifica* (Brazhnikova *in* Brazhnikova et al., 1967), tangential section, specimen SFA, no. 0076/003, Spl. R76; (7, 8, 9, 18) *Ikensieformis* aff. *mirifica* (Brazhnikova *in* Brazhnikova et al., 1967), (7) tangential section, specimen SFA, no. 0072/006, Spl. R72, (8) tangential section, specimen SFA, no. 0074/001, Spl. R74; (9) weakly oblique section, specimen SFA, no. 0057/003, Spl. R57; (18) axial section, specimen SFA, no. 0075/007, Spl. R75; (11, 12) *Ikensieformis* sp., (11) tangential section, specimen SFA, no. 0076/0010, Spl. R76, (12) oblique section, specimen SFA, no. 0085/001, Spl. R85; (13–17) *Ikensieformis persiaensis* Kulagina & Fassihi n. sp., (13) holotype, near axial section, specimen SFA, no. 0071/0025, Spl. R71, (14) tangential section, specimen SFA, no. 0071/0026, Spl. R71, (15) oblique median section, specimen SFA, no. 0071/0027, Spl. R71, (16) axial oblique section, specimen SFA, no. 0071/0028, Spl. R71, (17) axial oblique section, specimen SFA, no. 0069/0011, Spl. R69.

**FIGURE 8:**
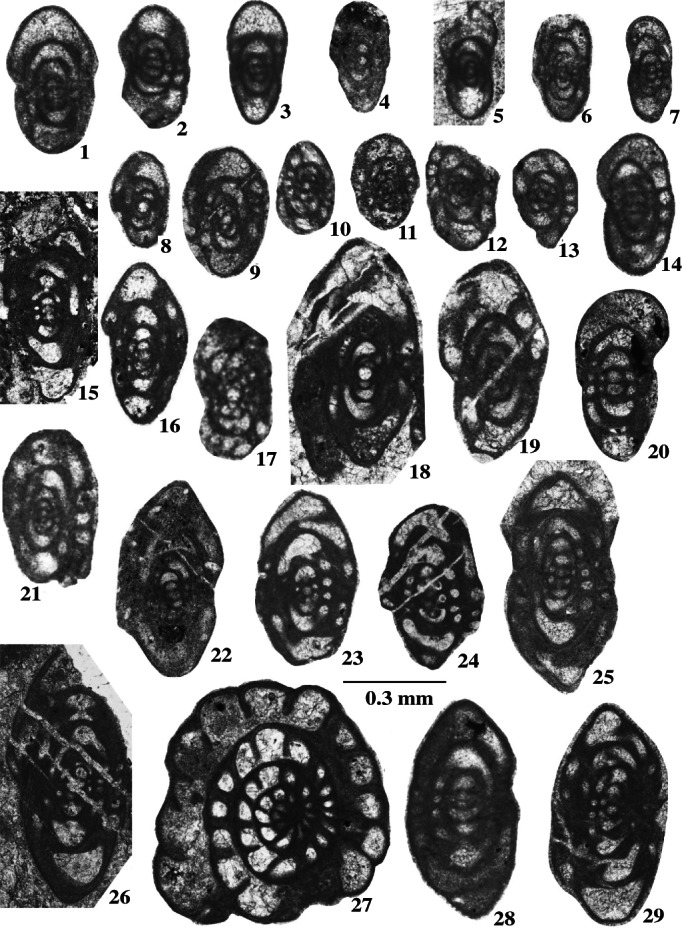
Selected late Serpukhovian–earliest Bashkirian? Foraminifers belonging to the family Eostaffellidae Mamet *in* Mamet et al. (1970). (1) *Eostaffellina paraprotvae* (Rauser‐Chernousova, 1948b), axial section, specimen SFA, no. 0071/0029, Spl. R71; (2) *Eostaffellina* sp., oblique section, specimen SFA, no. 0071/0030, Spl. R71, (3) *Eostaffella* aff. *grozdilovae* Maslo & Vachard, 1997, axial section, specimen SFA, no. 0071/0031, Spl. R71; (4) *Eostaffella* cf. *cooperi* (Zeller, 1953), axial section, specimen SFA, no. 0059/003, Spl. R59; (5) *Eostaffella* ex gr. *postmosquensis* Kireeva *in* Rauser‐Chernousova (1951), axial section, specimen SFA, no. 0071/0032, Spl. R71, (6) *Eostaffella postmosquensis* Kireeva *in* Rauser‐Chernousova (1951), axial section, specimen SFA, no. 0079/008, Spl. R79; (7) *Plectomillerella* cf. *excavata* (Niko, 1987), tangential‐oblique section, specimen SFA, no. 0076/004, Spl. R76; (8) *Eostaffella settella* Ganelina, 1951, axial section, specimen SFA, no. 0072/007, Spl. R72; (9) *Plectostaffella* cf. *acuminulata* Postojalko, 1990, axial section, specimen SFA, no. 0071/0034, Spl. R71; (10, 11) *Plectostaffella asymmetrica* Brazhnikova & Vdovenko *in* Aizenverg et al. (1983), (10) axial section, specimen SFA, no. 0074/006, Spl. R74, (11) median section, specimen SFA, no. 0079/008, Spl. R79; (12, 13) *Plectostaffella* cf. *uinskaja* Rumjanzeva *in* Kulagina et al. (1992), (12) incomplete axial section, specimen SFA, no. 0074/003, Spl. R74, (13) median section, specimen SFA, no. 0071/0035, Spl. R71; (14) *Plectostaffella* ex gr. *varvariensis* (Brazhnikova & Potievskaja, 1948), tangential section, specimen SFA, no. 0075/002, Spl. R75; (15) *Eostaffella* cf. *constricta* Ganelina, 1951, tangential section, specimen SFA, no. 0059/004, Spl. R59, (16) *Eostaffella constricta* Ganelina, 1951, almost axial section, specimen SFA, no. 0069/006, Spl. R69; (17) *Plectostaffella* sp., oblique section, specimen SFA, no. 0071/009, Spl. R71; (18, 19) *Eostaffella parastruvei* (Rauser‐Chernousova, 1948a), axial sections, (18) specimen SFA, no. 0070/004, Spl. R70, (19) specimen SFA, no. 0070/005, Spl. R70; (20) *Eostaffella* cf. *infulaeformis* (Ganelina, 1951), tangential section, specimen SFA, no. 0049/003, Spl. R49; (21) *Eostaffella* ex gr. *mosquensis* Vissarionova, 1948, axial section, specimen SFA, no. 0071/001, Spl. R71; (22–29) *Eostaffella igoi* Niko, 1987, (22) axial section, specimen SFA, no. 0076/006, Spl. R76, (23) almost axial section, specimen SFA, no. 0071/002, Spl. R71, (24) tangential section, specimen SFA, no. 0071/003, Spl. R71, (25) tangential section, specimen SFA, no. 0076/007, Spl. R76, (26) incomplete axial section, specimen SFA, no. 0069/001, Spl. R69, (27) median section, specimen SFA, no. 0069/002, Spl. R69, (28) tangential section, specimen SFA, no. 0075/003, Spl. R75, (29) oblique section, specimen SFA, no. 0076/008, Spl. R76.

**FIGURE 9:**
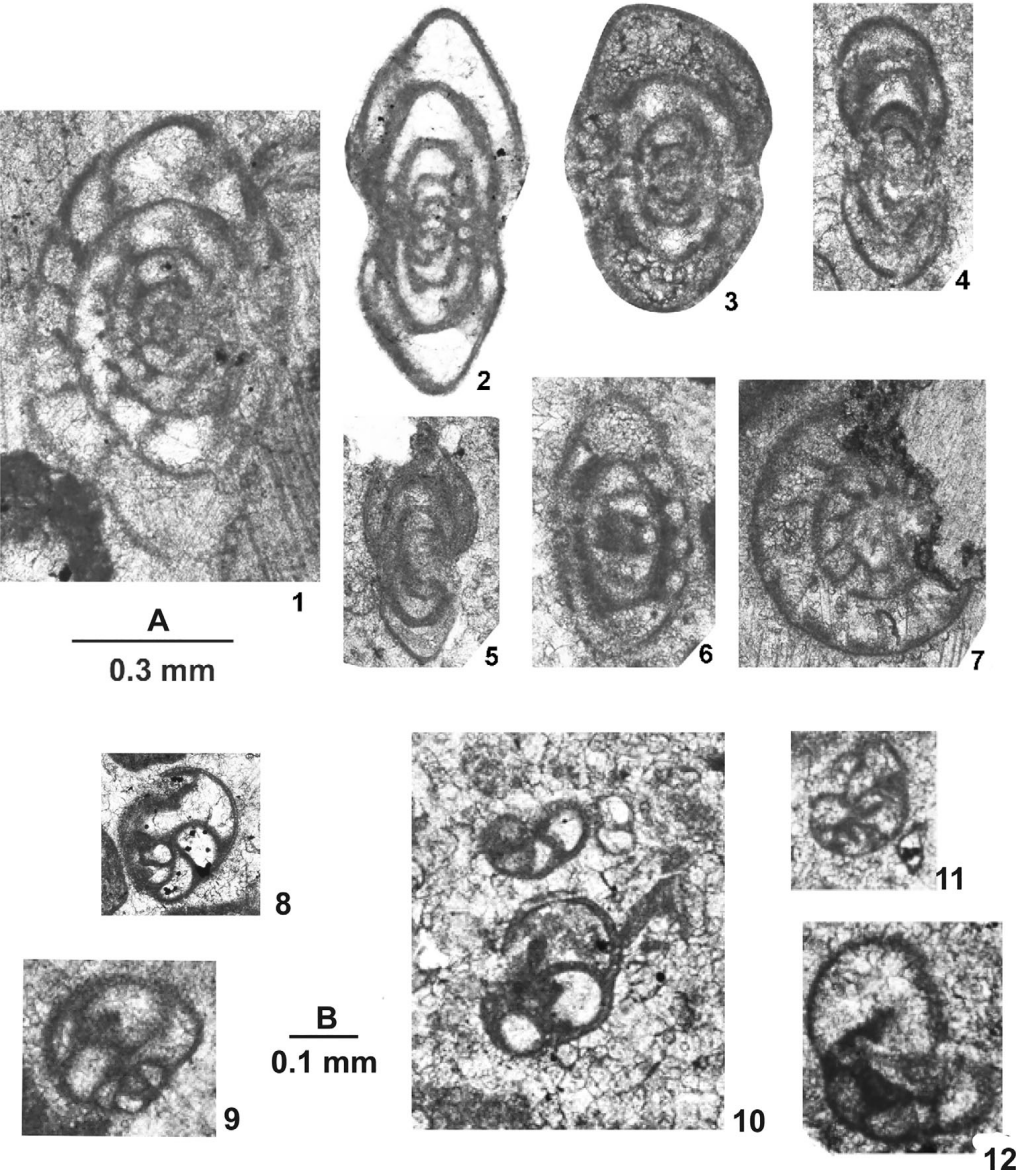
Selected late Serpukhovian–earliest Bashkirian? Foraminifers belonging to the order Staffellida Miklukho‐Maclay, 1949 and family Globivalvulinidae Reitlinger, 1950 of the Order Palaeotextulariida Hohenegger & Piller, 1975, from the Asad Abad II section. Spl. = sample number. Figures 1–7: Scale bar A; Figures 8–12: Scale bar B. (1) *Pseudoendothyra* sp., oblique‐median section, specimen SFA, no. 0071/004, Spl. R71; (2, 5) *Parastaffella* aff. *bona* (Rosovskaya, 1963), (2) axial section, specimen SFA, no. 0075/004, Spl. R75, (5) axial section, specimen SFA, no. 0076/009, Spl. R76; (3) *Pseudoendothyra* cf. *globosa* Rosovskaya, 1963, subaxial section, specimen SFA, no. 0071/005, Spl. R71; (4) *Parastaffella utkaensa* (Postojalko, 1990), axial section, specimen SFA, no. 0071/006, Spl. R71; (6, 7) *Parastaffella* sp., (6) tangential section, specimen SFA, no. 0071/007, Spl. R71, (7) median section, specimen SFA, no. 0071/008, Spl. R71; (8, 9) *Globivalvulina moderata* Reitlinger, 1949, (8) oblique axial section, specimen SFA, no. 0069/002, Spl. R69; (9) near axial section, specimen SFA, no. 0070/002, Spl. R70; (10, 12) *Globivalvulina* ex gr. *bulloides* (Brady, 1876), (10) (upper photo), median section, incomplete test, specimen SFA, no. 0070/001, Spl. R70, (lower photo), median section, specimen SFA, no. 0070/003, Spl. R70, (12) oblique axial section, specimen SFA, no. 0071/0022, Spl. R71; (11) *Globivalvulina* sp., sagittal section, specimen SFA, no. 0071/0021, Spl. R71.

Foraminifers are represented by an unusual biofacies dominated by encrusting forms, various endothyrids, palaeotextulariids, and ozawainellids. Representatives of *Ikensieformis* Orlova, 1997 and *Eostaffella* Rauser‐Chernousova, 1948a of the family Eostaffellidae Mamet *in* Mamet et al. (1970) (order Ozawainellida Solovieva, 1980) prevail. Archaediscidae Cushman, 1928 is very rare. The successive occurrence of taxa in the section allows four foraminiferal assemblages to be proposed (Figure 3), and the main foraminiferal taxa are illustrated in Figures 4, 5, 6, 7, 8, 9.

#### 3.2.1 Assemblage with *Biseriella minima* and *Eostaffellina paraprotvae*

**FIGURE 10:**
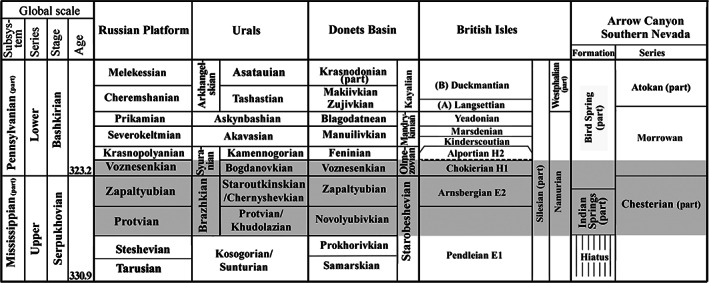
Correlation of the regional subdivisions of Russia (Alekseev et al., 2022; Postanovleniya, 2003), Donets Basin (Nemyrovska, 2017; Poletaev et al., 2013), British Isles (Cózar & Somerville, 2014; Richards, 2013), and Arrow Canyon, Southern Nevada (Brenckle et al., 2019; Groves et al., 1999)

**FIGURE 11:**
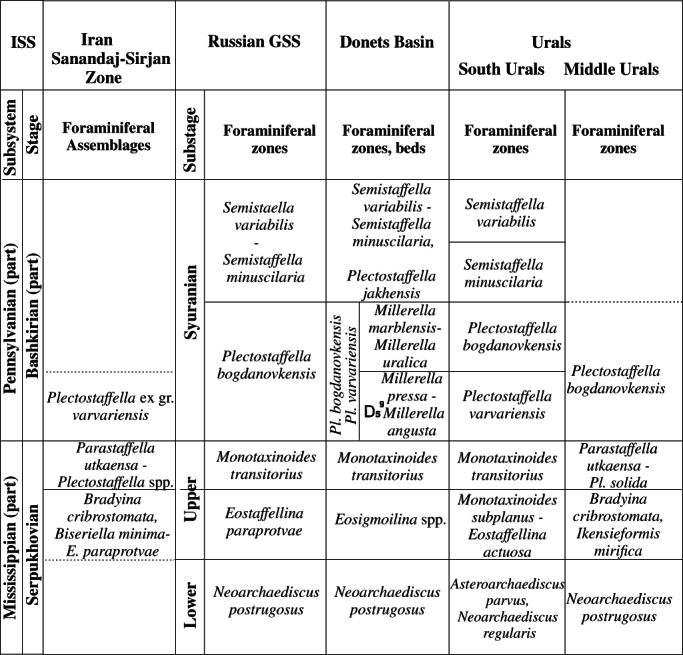
Correlation of the foraminiferal‐based biozonal schemes at the upper Serpukhovian–lower Bashkirian in Iran (Sanandaj–Sirjan zone), Russian general stratigraphic scale (Alekseev et al., 2022; Postanovleniya, 2003), Donets Basin (Nemirovska, 2017; Poletaev et al., 2013); south Urals (Kulagina, Nikolaeva, Pazukhin, & Kochetova, 2014; Nikolaeva et al., 2017); and middle Urals (Postojalko, 1990; Stepanova, 2018). *Biseriella minima*‐*E*. *paraprotvae*, *B. minima*‐*Eostaffellina paraprotvae*; *Pl*. *bogdanovkensis*, *Plectostaffella bogdanovkensis*; *Pl*. *solida*, *Plectostaffella solida*; *Pl*. *varvariensis*, *Plectostaffella varvariensis*

**FIGURE 12:**
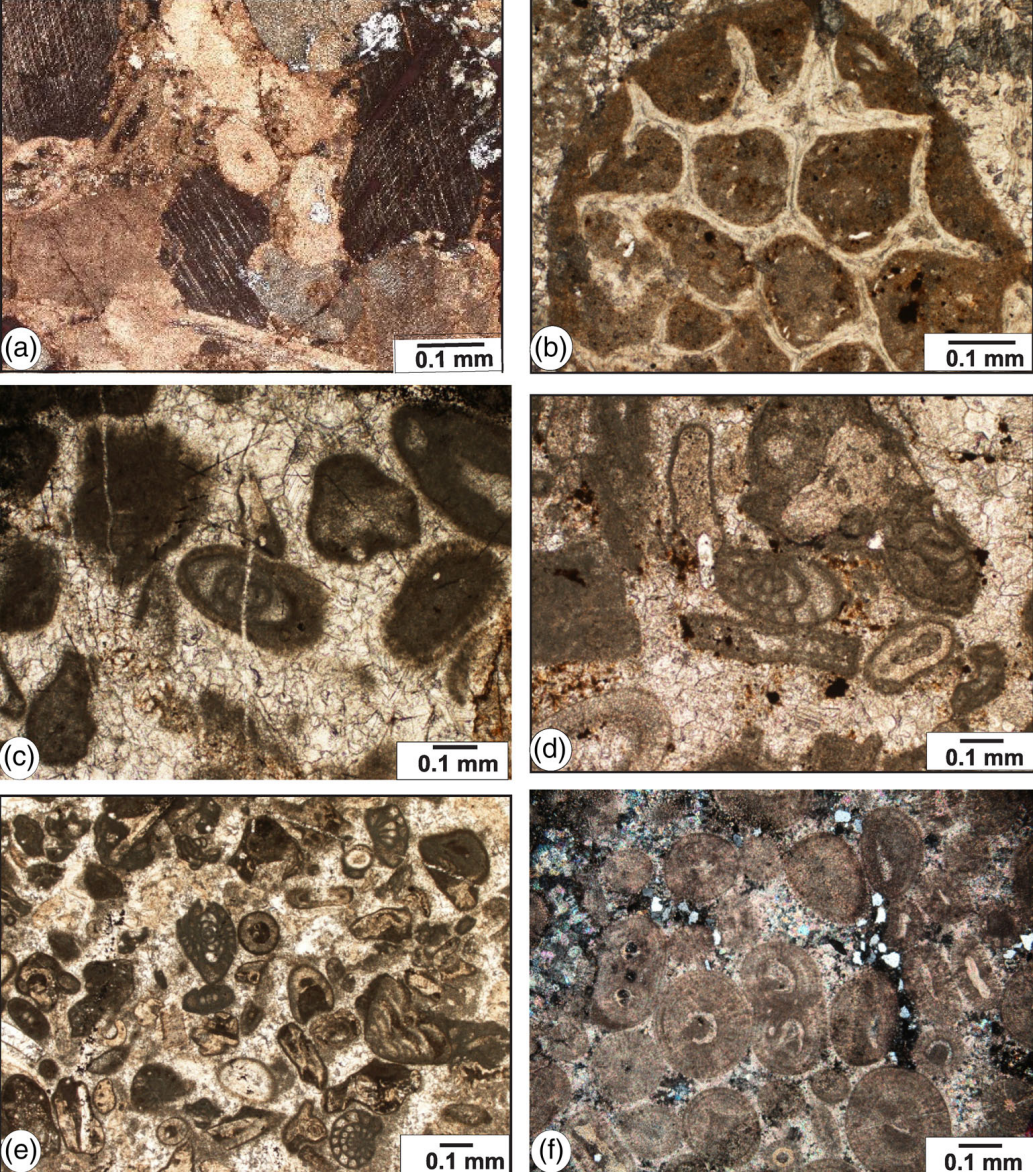
Photomicrographs of microfacies of the Ghaleh Formation in the Asad Abad II section in the Shahreza area. (a) Crinoidal packstone (MF1), Spl. R3; (b) Bioclastic packstone‐grainstone with bryozoans (MF2), Spl. R64; (c) Coated bioclastic grainstone with foraminifers (MF3), Spl. R4; (d) Bioclastic grainstone with foraminifers (MF4), Spl. R44; (e) Bioclastic grainstone (MF4), Spl. R69; (f) Oolitic grainstone (MF5), Spl. R45.

**FIGURE 13:**
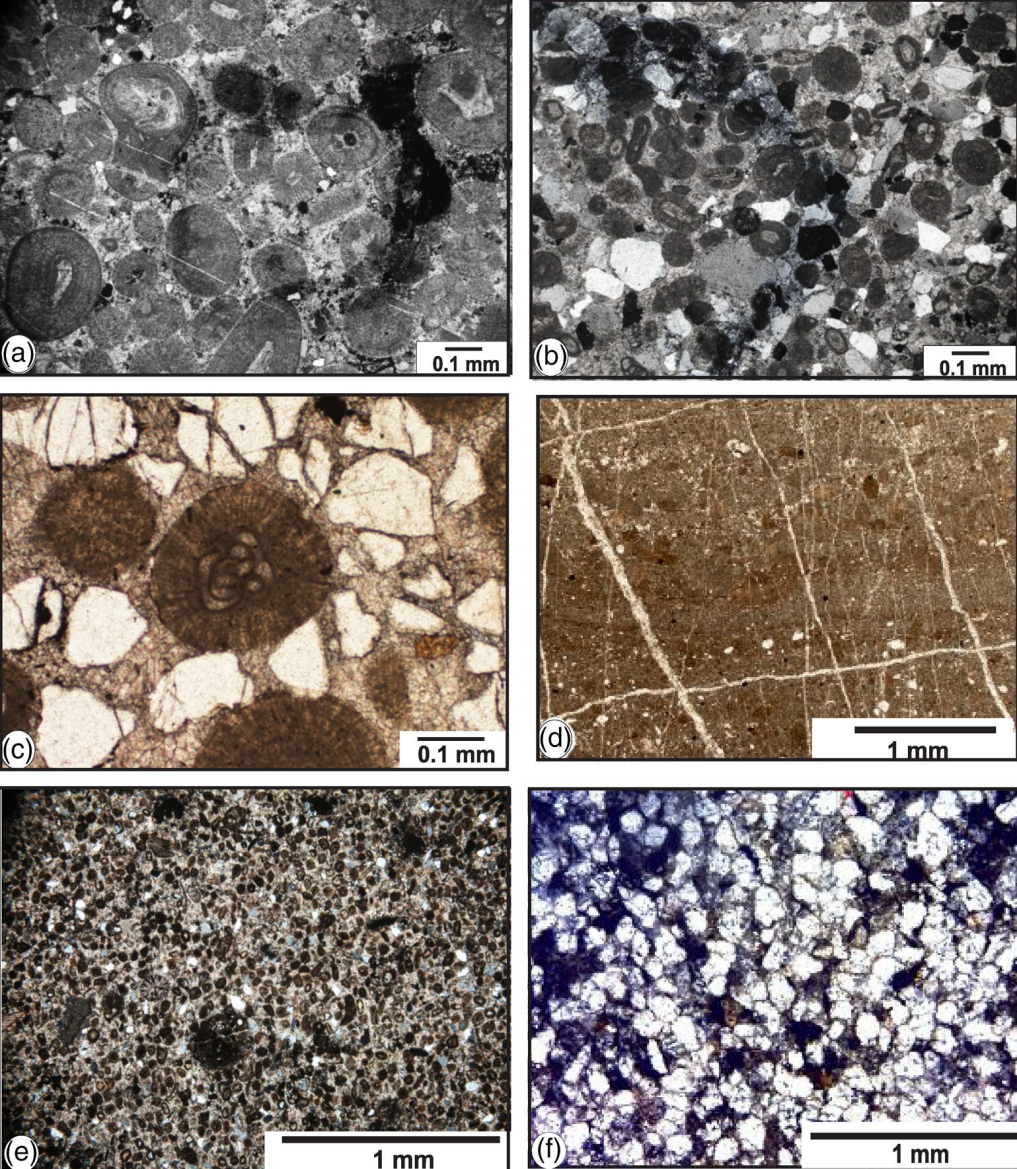
Photomicrographs of microfacies of the Ghaleh Formation in the Asad Abad II section in the Shahreza area. (a) Oolitic bioclastic grainstone with archaediscid foraminifers in ooid cores (MF5), Spl. R4.1; (b) Sandy oolitic grainstone with foraminifers (MF5), Spl. R77; (c) Sandy oolitic grainstone with *Pseudoglomospira* sp. (MF5), Spl. R81; (d) Mudstone‐Wackestone (MF6), Spl. R6; (e) Peloidal grainstone (MF7), Spl. R56; (f) Quartz arenitic sandstone (MF8), Spl. R83.

This assemblage was found in limestone beds with a total thickness of ca. 112 m (samples R4–R68). It is defined as the interval from the first occurrence of *Biseriella minima* (Reitlinger, 1950) to the first occurrence of *Bradyina cribrostomata* Rauser‐Chernousova & Reitlinger *in* Rauser‐Chernousova and Fursenko (1937). The first discoveries of foraminifers are associated with the oolitic limestone from the lower part of Unit 3 (sample R4, Figure 4). This assemblage consists of *Neoarchaediscus regularis* (Suleimanov, 1948) (Figure 4‐1), *Eostaffella* cf. *postmosquensis* Kireeva *in* Rauser‐Chernousova et al. (1951) (Figure 4‐11), and other species characterizing the upper Viséan‐Serpukhovian interval (see Figure 3). The most informative species is *B*. *minima* (Figure 4‐13) with well‐developed aperture septa in the last two chambers. Such forms are characteristic of the upper Serpukhovian and Bashkirian (e.g., Kulagina et al., 1992; Kulagina, Nikolaeva, Pazukhin, & Kochetova, 2014; Postojalko, 1990; Stepanova, 2018). The assemblage also contains *Eostaffellina paraprotvae* (Rauser‐Chernousova, 1948b) (Sample R34, Figure 4‐5), *Ikensieformis* aff. *mirifica* (Sample R57), and *Semiendothyra excellens* (Sample R63).

In the type area of the Serpukhovian, *E*. *paraprotvae* appears in the uppermost part of the Steshevian (Gibshman, 2003; Kabanov et al., 2016) or in the lower part of the Steshevian (Gibshman et al., 2009); and continues to the lower Protvian. In the eastern South Urals *E*. *paraprotvae* is characteristic of the Khudolazian regional Substage (Kulagina et al., 2009; Pazukhin et al., 2010). In the Middle Urals, Mariinsky Log Section, this species appears earlier; in the lower Serpukhovian, Kosogorian regional Substage (Nikolaeva et al., 2020, text‐figures 5, 7b). In the Pre‐Caspian Depression, *Semiendothyra* sp. is shown from the Protvian (Brenckle & Collins, 2017), and *S*. *excellens* first appears in the upper Zapaltyubian (Zaitseva & Klenina, 2008). In the Cantabrian Mountains, northern Spain *Semiendothyra* spp. including *S*. *excellens* appear somewhat above the Tarusian/Steshevian boundary (Cózar et al., 2016, 2019). In Turkey, *S*. *excellens* is shown from the top of the upper Viséan; in the *Endothyranopsis* (*Reitlingeropsis*) cf. *sphaerica‐Biseriella parva* Zone (Sample 84) (however in Figure 5, it is illustrated from Sample 27) (Demirel & Altıner, 2016). *Semiendothyra* ex gr. *excellens* is recorded from the uppermost Viséan in the Nashui (Naqing) section (Groves et al., 2012) and in Montagne Noire (France), *Semiendothyra excellens* was recorded from the biozone E, the uppermost biozone of the early Brigantian (Vachard et al., 2016). The presence of this species, and the appearance of *E*. *paraprotvae* (Rauser‐Chernousova, 1948b) does not contradict a late Serpukhovian (specifically Protvian) age, of this interval.

#### 3.2.2 Assemblage with *Bradyina cribrostomata*

This assemblage with a thickness of about 14 m (samples R69 and R70) is defined as the interval from the first occurrence of *B*. *cribrostomata* Rauser‐Chernousova & Reitlinger *in* Rauser‐Chernousova and Fursenko (1937) (Figure 6‐16) and *Parastaffella utkaensa* (Postojalko, 1990). The most informative species are *B*. *cribrostomata* (Figure 6‐16), *Globivalvulina moderata* Reitlinger, 1949 (Figure 9‐8 and ‐9); and *Globivalvulina* ex gr. *bulloides* (Brady, 1876) (Figure 9‐10 and ‐12). The presence of large tests of *Ikensieformis* spp., *Eostaffella igoi* Niko, 1987 (Figure 8‐26 and ‐27); *E*. *parastruvei* Rauser‐Chernousova, 1948a (Figure 8‐18 and ‐19), and *Ikensieformis persiaensis* sp. nov. (Figure 7‐17) is worth mentioning.

*B*. *cribrostomata* is a typical species for the Protvian and its analogues (e.g., Aizenverg et al., 1983; Stepanova, 2018). This species is currently proposed to be considered as an index species of the Protvian and Khudolasian substages of the Urals (Alekseev et al., 2022; Ponomareva et al., 2016; Stepanova, 2018).

The late Serpukhovian age of these beds is confirmed by the appearance of species of *Globivalvulina* (*G*. *moderata* group) with a discontinuous, central light‐coloured layer of the wall (samples R69 and R70; Figure 9‐8 and ‐9). *G*. *moderata* was first described from the Bashkirian (Lakly Section, South Urals). Later, this species was also found in the older beds, that is, in the late Serpukhovian sections of Eurasia and North America. According to Brenckle (2005, p. 49) synthesizing the data of Harris et al. (1997) and Mizuno (1997), ‘thin‐walled globivalvulinins belonging or related to *G*. *moderata* appeared in the Late Mississippian throughout the Palaeotethys and Arctic North America, and migrated into sub‐Arctic North America during the Early Pennsylvanian’.

*Globivalvulina* ex gr. *moderata* is characteristic of the upper Serpukhovian of the Donets Basin (Aizenverg et al., 1983), Urals (Postojalko, 1990; Stepanova, 2018), Pre‐Caspian Basin (Brenckle & Collins, 2017), North America (Groves et al., 1992; Harris et al., 1997; Mamet et al., 1993). However, in North Africa this species was recorded in the early Serpukhovian (bed M of the Tinguiz Remz Section of the Saharan Tindouf Basin) (Cózar, Medina Varea, et al., 2014). According to Cózar and Somerville (2012), the primitive species of *Globivalvulina* probably appeared in the lower part of the Serpukhovian, that is, in the Steshevian Substage, where they were very rare.

The first appearance of *G*. *moderata* (*= G*. *bulloides*) in Arctic Alaska is below the Mid‐Carboniferous boundary (Baesemann et al., 1997; Harris et al., 1997). According to Brenckle and Milkina (2003), *Globivalvulina* of the *G*. *bulloides* group is one of the species, that help to recognize the Protvian Horizon in the Tengiz Platform in Kazakhstan. In the South Urals, the first appearance level of *G*. *bulloides* is recorded in the Protvian, in the *Monotaxinoides subplanus‐Eostaffellina actuosa* Zone in the Muradymovo section (Kulagina, Nikolaeva, Pazukhin, & Kochetova, 2014), but this single specimen is more likely to belong to *G*. *moderata* based on the wall structure. *G*. *bulloides* is characteristic of the uppermost Serpukhovian of the Urals and Pre‐Caspian Basin (Stepanova & Kucheva, 2006; Zaitseva & Klenina, 2008). In Western Europe, this species also usually appears in the late Serpukhovian, but in North Africa, it is a marker of the base of the Bashkirian (Cózar et al., 2011).

The Assemblage with *B*. *cribrostomata* can be correlated with the *E*. *paraprotvae* Zone of the General Stratigraphic Scale of Russia, *B*. *cribrostomata–Ikensieformis mirifica* Zone of the eastern slope of the Urals (Stepanova, 2018), and the *E*. *paraprotvae–B*. *cribrostomata* Zone of Turkey (Altiner & Özgül, 2001). This assemblage is also similar to the assemblage of the *Eostaffella* ex gr. *ikensis*–*Eostaffella postmosquensis* Zone of the Central Taurides, Turkey but this zone correlates with the Zapaltyubian Substage (Atakul‐Özdemir et al., 2011).

#### 3.2.3 Assemblage with *Parastaffella utkaensa* – *Plectostaffella* spp.

This assemblage, with a thickness of about 27 m, is defined as the interval from the first occurrence of *P*. *utkaensa* and *Plectostaffella* sp. to the first occurrence of *Plectostaffella* ex gr. *varvariensis* (Brazhnikova & Potievskaja, 1948) (samples R71–R74). The strata bearing these faunas were initially assigned to the lowermost part of the Bashkirian (Voznesenkian), corresponding to the *Plectostaffella jakhensis‐Eostaffella pseudostruvei* Zone (e.g., Fassihi, 2017; Fassihi & Shirezadeh, 2018). Many species found in this assemblage continue from the underlying beds. The most significant additions to this assemblage are *Bradyina concinna* Reitlinger, 1950 (Figure 6‐15); *Plectostaffella* sp. (Figure 8‐17); *Pl*. cf. *acuminulata* Postojalko, 1990 (Figure 8‐9); *Pl*. cf. *uinskaja* Rumjanzeva *in* Kulagina et al. (1992) (Figure 8‐12 and ‐13); *Pl*. *asymmetrica* Brazhnikova & Vdovenko *in* Aizenverg et al. (1983) (Figure 8‐10), and *P*. *utkaensa* Postojalko, 1990 (Figure 9‐4). The assemblage also includes encrusting forms; most often *Scalebrina* sp. (Figure 5‐22), *Pseudoglomospira subquadrata* (Figure 5‐20), *Pseudoendothyra* cf. *globosa* Rosovskaya, 1963 (Figure 9‐3), and representatives of the Endothyridae and Eostaffellidae.

*Plectostaffella acuminulata* and *P*. *utkaensa* are characteristic of the uppermost Serpukhovian (Staroutkinskian Substage) of the western subregion of the Urals (Alekseev et al., 2022; Stepanova, 2018). Records of *B*. *concinna* support the late Serpukhovian–early Bashkirian age of the host beds (Brenckle & Milkina, 2003; Kulagina et al., 1992; Popova & Reitlinger, 1973; Stepanova & Kucheva, 2006). The typical upper Viséan taxa sach a*s Ikensieformis ikensis* (Vissarionova, 1948) (Figure 7‐1 and ‐3) and *I*. *proikensis* (Rauser‐Chernousova, 1948b) (Figure 7‐10) are also present in this assemblage. A taxon similar *to I*. *mirifica* (Brazhnikova *in* Brazhnikova et al., 1967) is also widespread (Sample R75) (*I*. aff. *mirifica*; samples R72 and R74). Species characteristic of the upper Serpukhovian and lower Bashkirian, such as *E*. *paraprotvae* and *Eostaffella* ex gr. *postmosquensis* Kireeva *in* Rauser‐Chernousova et al. (1951) are present.

Based on the appearance of the genus *Plectostaffella* and other taxa, the assemblage with *P*. *utkaensa* and *Plectostaffella* spp. can be compared to the *Plectostaffella acuminulata–P*. *utkaensa* foraminiferal zone of the western subregion of the Urals which characterizes the Staroutkinskian Regional Substage. The latter is correlated with the Chernyshevkian Regional Substage of the eastern subregion of the Urals with *Plectostaffella varvariensiformis* foraminiferal Zone (Alekseev et al., 2022; Stepanova & Kucheva, 2006), as well as with the Zapaltyubian Regional Substage of the Donets Basin.

#### 3.2.4 Assemblage with *Plectostaffella* ex gr. *varvariensis*

The beds, with a thickness of about 26 m (samples R75–R85), are characterized by the first appearance of *Plectostaffella* ex gr. *varvariensis* (Brazhnikova & Potievskaja, 1948). The top of the interval is unconformably overlain by sediments of the same formation (Unit 6) of the uppermost Bashkirian–lowermost Moscovian [the Melekessian–Vereian *Tikhonovichella tikhonovichi‐Profusulinella* (*Depratina*) *prisca‐Aljutovella* spp. Zone] (Fassihi et al., 2017).

The assemblage with *Plectostaffella* ex gr. *varvariensis* is rather impoverished and similar to that of the underlying assemblage. *Plectostaffella* ex gr. *varvariensis* (Figure 8‐14); *Plectomillerella* cf. *excavata* (Niko, 1987) (Figure 8‐7); and *Parastaffella* aff. *bona* Rosovskaya, 1963 (Figure 9‐2 and ‐5) and typical *Eostaffella postmosquensis* (Figure 8‐6) first appear at this level.

The assemblage also includes *Plectostaffella asymmetrica* (Figure 8‐11), *Ikensieformis* aff. *mirifica* (Figure 7‐18), and *E*. *igoi* (Figure 8‐22, ‐25, ‐28 and ‐29) emerging from the underlying beds. Large *Ikensieformis* (*I*. *persiaensis*, and *I*. *proikensis*) do not continue into this zone.

An analysis of the distribution of *Plectostaffella* species in different regions by Cózar and Somerville (2014, 2021) showed that species of the *Pl*. *varvariensis* group appear at different stratigraphic levels, in the interval from the upper Serpukhovian to the lower Bashkirian, inclusive.

In the Urals and the Middle Tien Shan, in the upper Serpukhovian deposits, there are primitive *Plectostaffella* with a slight deviation of the winding axis in the last whorls, such as *Plectostaffella primitivа* Rumjanzeva *in* Kulagina et al. (1992) [= *Plectostaffella varvariensis* (Brazhnikova & Potievskaja, 1948) *in* Nikolaeva et al., 2009], *Pl*. ex gr. *P*. *primitiva in* Nikolaeva et al. (2017) [=*Pl*. ex gr. *varvariensis in* Kulagina et al., 1992, pl. 3, figure 31], *Pl*. *posochovae* Rumjanzeva *in* Kulagina et al., 1992 and other species of this group and also *Pl*. *reitlingeri* Groves, 1988 (Ponomareva, 2004), and *Pl*. *varvariensiformis* Brazhnikova & Vdovenko *in* Aizenverg et al. (1983) (Stepanova & Kucheva, 2006).

In the South Urals, in the Muradymovo section, the first appearance of *Pl*. *varvariensis* is marked above the first appearance of the conodont species *Declinognathodus noduliferus* in beds with mostly Serpukhovian foraminiferal assemblage (Kulagina, Nikolaeva, Pazukhin, & Kochetova, 2014; Kulagina, Nikolaeva, & Pazukhin 2014). In the Bolshoi Kizil section, *Pl*. *varvariensis* appears almost at the same level *as Declinognathodus noduliferus* (Kulagina et al., 2009). A similar succession is described in the Middle Urals, in the Gostinsky section: the base of the *Pl*. *varvariensis* Zone coincides with the early *D*. *noduliferus* conodont Zone (Ponomareva, 2004).

On the other hand, *Pl*. *varvariensis* was indicated from the Zapaltyubian Regional Substage of the Donets Basin (Aizenverg et al., 1983), however, a typical specimen with a noticeable displacement of the last whorl is illustrated from the Voznesenkian Regional Substage (Limestone D_7_). Shells of *Pl*. *varvariensis* from equivalents of the Protvian (uppermost) of the Vegas de Sotres section (Cózar et al., 2016) are more symmetrical than the holotype. Sheng et al. (2018) reported *Pl*. *varvariensis* from the uppermost Serpukhovian in China.

In this study and in the uppermost of Unit 5, the typical Bashkirian *Plectostaffella* with a distinct whorl asymmetry was not found. Nevertheless, because of the presence of *Pl*. ex gr. *varvariensis*, the strata bearing these faunas were initially assigned to the lowermost part of the Bashkirian (Voznesenkian). As a consequence, due to lack of the sufficient data in this study, the lowermost Bashkirian (Voznesenkian) cannot be exactly attributed to the upper part of Unit 5 of the Ghaleh Formation. We can only suggest the presence of the lowest Bashkirian in the Asad‐Abad Section II based on the presence of *D*. *noduliferus* in the nearby Asadabad section (Boncheva et al., 2007). This can be established precisely only by studying the conodonts from the Asad Abad II section.

## 4 DISCUSSION

The late Serpukhovian age of units 3 and 4, and the lower part of Unit 5 of the Ghaleh Formation is based on foraminifers that are characteristic of the late Serpukhovian in many sections world‐wide: East European Platform (e.g., Gibshman, 2003; Kabanov et al., 2016), Donets Basin (Aizenverg et al., 1983; Vachard & Maslo, 1996), the Urals (Kulagina et al., 1992; Kulagina, Nikolaeva, Pazukhin, & Kochetova, 2014; Nikolaeva et al., 2017; Postojalko, 1990; Stepanova, 2018; Stepanova & Kucheva, 2006), Pre‐Caspian Basin, Kazakhstan (Akhmetshina et al., 2007; Brenckle & Collins, 2017; Brenckle & Milkina, 2003; Collins & Brenckle, 2017; Zaitseva & Klenina, 2008), British Isles (Cózar & Somerville, 2014), Tien Shan (Kulagina et al., 1992; Rumjanzeva, 1970), and North Africa (Cózar et al., 2011; Cózar, Vachard, et al., 2014). However, the taxonomic composition of the studied foraminifers has its own characteristics.

A comparison of the upper Serpukhovian foraminiferal assemblages of the Asad‐Abad II section with the late Serpukhovian assemblage of Mid‐Carboniferous boundary stratotype shows considerable differences. The late Serpukhovian foraminiferal assemblage in the Arrow Canyon section (Nevada, USA) contains representatives of the Archaediscida [*Neoarchaediscus*, *Brenckleina*, *Eosigmoilina*, and *Betpakodiscus*] (Brenckle, 1991; Brenckle et al., 1997, 2019; Lane et al., 1999). The listed species are not found in the deposits of the Sanandaj–Sirjan Zone of Iran, with the exception of a few specimens of *Neoarchaediscus*. For comparison with the Serpukhovian‐type region, it is possible to use the data on the Tarusian, Steshevian, and Protvian regional substages. In the Stratigraphic Scale of the Russian Platform, the upper Serpukhovian includes the Protvian and Zapaltyubian regional substages and the lower Bashkirian corresponds to the Voznesenkian and Krasnopolyanian regional substages (Alekseev et al., 2004, 2022; Postanovleniya, 2003) (Figure 10). The Mid‐Carboniferous boundary in the Moscow Basin is marked by a gap, therefore, for the East European Platform, subdivisions of Donets Basin (Zapaltyubian and Voznesenkian) were used, where this interval is represented by a complete sequence (Aizenverg et al., 1978).

The Asad‐Abad II section contains taxa in common with the *E*. *paraprotvae* assemblage of the Protvian Regional Substage of the Moscow Region (Zaborie and Novogurovsky quarry sections) (Gibshman et al., 2009; Kabanov et al., 2009, 2016) such as *Janischewskina* ex gr. *delicata*, *Ikensieformis* aff. *mirifica*, and *E*. *paraprotvae*.

In the current stratigraphic scheme for the Donets Basin (Poletaev et al., 2013), the upper Serpukhovian corresponds to the Starobeshevian Regional Stage and includes the Prokhorivkian, Novolyubivkian, and Zapaltyubian regional substages (horizons). However, in the stratigraphic scheme of Nemyrovska (2017), the Prokhorivkian is an equivalent of the Steshevian, and Novolyubivkian corresponds to the Protvian of Eastern Europe. According to Poletaev et al. (2013), the Prokhorivkian and Novolyubivkian correspond to the *Eosigmoilina* spp. foraminiferal Zone, and Zapaltyubian to the *Monotaxinoides transitorius* Zone. In the Asad Abad II section we did not find eosigmoilinids. Of lasiodiscids, only *Howchinia* cf. *beianensis* Shen & Wang, 2017 (Sample R18) and *H*. *gibba minima* Vdovenko, 1960 (Sample R71) were found. Species in common with the upper Serpukhovian assemblages of the Donets Basin are mainly representatives of the Family Eostaffellidae, such as *Eostaffella postmosquensis*, *E*. *paraprotvae*, and *Ikensieformis* spp. In the Donets Basin, records of *Globivalvulina* begin from the Zapaltyubian (Aizenverg et al., 1983).

The most species‐diverse foraminiferal assemblage was identified in the assemblage with *P*. *utkaensa* and *Plectostaffella* spp. This assemblage contains species in common with the Staroutkinskian and Chernyshevkian regional substages of the Urals, which are compared with those of the Zapaltyubian. The first appearance of *Plectostaffella* distinguishes this zone from the underlying deposits.

The assemblage with *B*. *cribrostomata* and the assemblage with *P*. *utkaensa* and *Plectostaffella* spp. can be recognized as an eostaffellid‐parastaffellid biofacies. This biofacies is characterized by the presence of numerous encrusting taxa, representatives of the family Eostaffellidae, diverse *Eostaffella*, especially abundant *Ikensieformis*, frequent *Parastaffella*, and rare *Globivalvulina*. Specimens of Archaediscida are very rare. This foraminiferal biofacies is associated with the shallowest water conditions and high‐energy environment.

The youngest foraminiferal fauna identified in this research is the assemblage with *Plectostaffella* ex gr. *varvariensis*. This assemblage is close to the *Plectostaffella varvariensis* Zone (Kulagina, Nikolaeva, Pazukhin, & Kochetova, 2014; Ponomareva, 2004) and possibly correlates with the lower part of the *Plectostaffella bogdanovkensis* Zone of the General Stratigraphic Scale of Russia (Figure 11).

## 5 SYSTEMATIC PALAEONTOLOGY

Two fusulinoid forms, that is, *E*. *igoi* and *Ikensieformis* aff. *mirifica* and the new species *I*. *persiaensis* are examined herein. For the systematics, the classification scheme of Rauser‐Chernousova et al. (1996), and Ginkel (2010) is principally followed.

The following abbreviations are used for the description of the fusulinoids: (No. of V) = number of volutions; (L) = length; (D) = diameter; (FR) = form ratio: L/D; (DP) = diameter of the proloculus; (TW) = thickness of the last wall.

Order OZAWAINELLIDA Solovieva, 1980

Family EOSTAFFELLIDAE Mamet *in* Mamet et al. (1970)

Genus *Ikensieformis* Orlova, 1997

Type species *Eostaffella ikensis* Vissarionova, 1948

*Ikensieformis persiaensis* Kulagina & Fassihi sp. nov.

Figure 7‐13 and ‐17

*Etymology*: After Persia, the ancient name of Iran, where the species was collected.

*Holotype*: Near axial section, specimen SFA, no. 0071/0025, Spl. R71, Figure 7‐13.

*Material examined* (*type series*): Paratypes: specimen SFA, no. 0071/0026, Spl. R71, [Figure 7‐14, tangential section]; specimen SFA, no. 0071/0027, Spl. R71, [Figure 7‐15, oblique median section]; specimen SFA, no. 0071/0028, Spl. R71, [Figure 7‐16, axial oblique section]; specimen SFA, no. 0069/0011, Spl. R69 [Figure 7‐17, axial oblique section].

*Repository*: The described specimen is numbered and housed in the senior author's personal collection with the prefix SFA.

*Description*: Test is medium to large, lenticular to rhomboidal in axial view, with keel and rounded periphery. Coiling is planispiral, involute, slightly loose, and chamber height gradually increases through growth, especially in the two last volutions. Umbilical depressions are absent. Spirotheca is thin inner and composed of primary tectum and undifferentiated protheca. In the inner volutions, the wall is covered by a secondary floor layer that merges into the low chomata.

*Measurements*: Measurements are given in Tables 1, 2, 3.

**TABLE 1:** Measurements of the described fusulinoids

Specimen	Figure	No. of V	L	D	FR	DP	TW
*Ikensieformis persiaensis*	Figure 7‐13	3	0.42	0.87	0.525	0.07	0.013
*I*. *persiaensis*	Figure 7‐14	4–4.5	0.51	0.95	0.536	‐	0.015
*I*. *persiaensis*	Figure 7‐15	3–4	0.49	0.82	0.598	‐	0.013
*I*. *persiaensis*	Figure 7‐16	3.5	0.40	0.80	0.5	‐	0.010
*I*. *persiaensis*	Figure 7‐17	4	0.47	0.88	0.534	‐	0.027
*Ikensieformis* aff. *mirifica*	Figure 7‐7	4	0.33	0.72	0.458	‐	0.020
*I*. aff. *mirifica*	Figure 7‐8	4	0.22	0.46	0.478	‐	0.010
*I*. aff. *mirifica*	Figure 7‐9	3–3.5	0.31	0.55	0.581	‐	0.010
*I*. aff. *mirifica*	Figure 7‐18	3–3.5	0.32	0.582	0.515	0.008	0.017
*Eostaffella igoi*	Figure 8‐22	6	0.3	0.58	0.517	‐	0.010
*E*. *igoi*	Figure 8‐23	4	0.24	0.49	0.489	0.015	0.010
*E*. *igoi*	Figure 8‐24	4	0.29	0.46	0.630	‐	0.005
*E*. *igoi*	Figure 8‐25	5	0.29	0.63	0.460	‐	0.031
*E*. *igoi*	Figure 8‐26	4	0.34	0.74	0.459	‐	0.020
*E*. *igoi*	Figure 8‐28	6	0.320	0.651	0.491	‐	0.020
*E*. *igoi*	Figure 8‐29	5	0.341	0.662	0.515	‐	0.020

Abbreviations: D, diameter; DP, proloculus diameter; FR, form ratio; L, length; No. of V, number of volutions; TW, wall thickness.

**TABLE 2:** Measurements of the radius vector of the described fusulinoids

Specimen	Figure	Radius vector					
		1	2	3	4	5	6
*Ikensieformis persiaensis*	Figure 7‐13	0.155	0.234	0.431	‐	‐	‐
*I*. *persiaensis*	Figure 7‐14	0.050	0.117	0.273	0.456	‐	‐
*I*. *persiaensis*	Figure 7‐15	0.061	0.320	0.410	‐	‐	‐
*I*. *persiaensis*	Figure 7‐16	0.130	0.260	0.410	‐	‐	‐
*I*. *persiaensis*	Figure 7‐17	0.050	0.130	0.230	0.440	‐	‐
*Ikensieformis* aff. *mirifica*	Figure 7‐7	0.070	0.160	0.250	0.351	‐	‐
*I*. aff. *mirifica*	Figure 7‐8	0.020	0.060	0.105	0.230	‐	‐
*I*. aff. *mirifica*	Figure 7‐9	0.075	0.160	0.267	‐	‐	‐
*I*. aff. *mirifica*	Figure 7‐18	0.052	0.088	0.194	0.282	‐	‐
*Eostaffella igoi*	Figure 8‐22	0.011	0.031	0.050	0.110	0.191	0.280
*E*. *igoi*	Figure 8‐23	0.020	0.081	0.140	0.24	‐	‐
*E*. *igoi*	Figure 8‐24	0.062	0.077	0.142	0.228	‐	‐
*E*. *igoi*	Figure 8‐25	0.010	0.051	0.070	0.220	0.310	‐
*E*. *igoi*	Figure 8‐26	0.020	0.050	0.151	0.241	0.373	‐
*E*. *igoi*	Figure 8‐28	0.041	0.093	0.124	0.206	0.279	0.321
*E*. *igoi*	Figure 8‐29	0.020	0.072	0.144	0.227	0.331	‐

**TABLE 3:** Measurements of the half‐length of the described fusulinoids

Specimen	Figure	Half‐length						
		1		2	3	4	5	6
*Ikensieformis persiaensis*	Figure 7‐13	0.065		0.143	0.210	‐	‐	‐
*I*. *persiaensis*	Figure 7‐14	0.052		0.091	0.180	0.252	‐	‐
*I*. *persiaensis*	Figure 7‐15	0.060		0.130	0.230	‐	‐	‐
*I*. *persiaensis*	Figure 7‐16	0.070		0.130	0.200	‐	‐	‐
*I*. *persiaensis*	Figure 7‐17	0.020		0.060	0.130	0.240	‐	‐
*Ikensieformis* aff. *mirifica*	Figure 7‐7		0.030	0.080	0.110	0.160	‐	‐
*I*. aff. *mirifica*	Figure 7‐8	0.020		0.050	0.070	0.110	‐	‐
*I*. aff. *mirifica*	Figure 7‐9	0.053		0.085	0.162	‐	‐	‐
*I*. aff. *mirifica*	Figure 7‐18	0.07		0.105	0.141	0.158	‐	‐
*Eostaffella igoi*	Figure 8‐22	0.010		0.031	0.040	0.071	0.080	0.150
*E*. *igoi*	Figure 8‐23	0.02		0.03	0.1	0.12	‐	‐
*E*. *igoi*	Figure 8‐24	0.031		0.052	0.070	0.139	‐	‐
*E*. *igoi*	Figure 8‐25	0.010		0.021	0.040	0.110	0.140	‐
*E*. *igoi*	Figure 8‐26	0.011		0.030	0.060	0.130	0.163	‐
*E*. *igoi*	Figure 8‐28	0.020		0.041	0.051	0.082	0.114	0.156
*E*. *igoi*	Figure 8‐29	0.031		0.062	0.103	0.144	0.173	‐

*Comparison*: *I*. *persiaensis* resembles *I*. *pespicabila* Grozdiliva & Lebedeva, 1954 in its shape and size. However, the present new species differs from *I*. *pespicabila* by having a lower number of volutions, the high last volution with an angular periphery, and a thick wall. In addition, owing to having a high free spiral, a smaller number of volutions with almost the same size in the inner volution, and a distinct keel, the described species can easily be distinguished from all known species of the genus *Ikensieformis*. Furthermore, this new species is found in the upper Serpukhovian.

*Occurrence and age*: Late Serpukhovian; samples R69, R71; Ghaleh Formation, Asad Abad II section, Sanandaj–Sirjan Zone, Iran.

*Ikensieformis* aff. *mirifica* (Brazhnikova in Brazhnikova et al., 1967)

Figure 7‐7, ‐9 and ‐18

*Material examined*: One axial, two tangential, and one weakly oblique sections illustrated. (Figure 7‐7) specimen SFA, no. 0072/006, Spl. R72; (Figure 7‐8) specimen SFA, no. 0074/001, Spl. R74; (Figure 7‐9) specimen SFA, no. 0057/003, Spl. R57; (Figure 7‐18) specimen SFA, no. 0075/007, Spl. R75.

*Description*: Test is medium, rhomboid to slender lenticular in axial view, with pointed periphery and convex to almost parallel lateral slopes. Coiling is planispiral and involute. Umbilical depressions are absent. Spirotheca is composed of a tectum and a dark and homogeneous protheca. Specimen no. 0074/001 Figure 7‐18 has endothyroid coiling of the initial whorl.

*Measurements*: Measurements are given in Tables 1, 2, 3.

*Comparison*: The current specimens resemble *I*. *mirifica* from the northern slope of the Ukrainian shield (Aizenverg et al., 1983; Brazhnikova et al., 1967) and from the eastern slope of the Middle Urals (Stepanova, 2018; Stepanova & Kucheva, 2006), based on its rhomboid to slender lenticular shape and lack of umbilical depressions. The Iranian forms, however, differ by having a larger diameter, more inflated shape, more loosely coiled test, and less pointed periphery. Specimen R75 also has an endothyroid first whorl. The current specimens can be distinguished from *I*. *persiaensis* by their smaller size and smaller form ratio.

*Occurrence and age*: Late Serpukhovian; samples R57, R72, R74, R75; Ghaleh Formation, Asad Abad II section, Sanandaj–Sirjan Zone, Iran

Genus *Eostaffella* Rauser‐Chernousova, 1948a

Type species *Staffella* (*Eostaffella*) *parastruvei* Rauser‐Chernousova, 1948a

*Eostaffella igoi* Niko, 1987

Figure 8‐22 and ‐29

*Material examined*: Seven sections illustrated. (Figure 8‐22) axial oblique section, specimen SFA, no. 0076/006, Spl. R76; (Figure 8‐23) axial section, specimen SFA, no. 0071/002, Spl. R71; (Figure 8‐24) tangential section, specimen SFA, no. 0071/003, Spl. R71; (Figure 8‐25) tangential section, specimen SFA, no. 0076/007, Spl. R76; (Figure 8‐26) incomplete axial section, specimen SFA, no. 0069/001, Spl. R69; (Figure 8‐27) median section, specimen SFA, no. 0069/002, Spl. R69; (Figure 8‐28) tangential section, specimen SFA, no. 0075/003, Spl. R75; (Figure 8‐29) oblique section, specimen SFA, no. 0076/008, Spl. R76.

*Description*: Test is large, lenticular to discoidal in axial view, with pointed periphery and rather straight to almost convex lateral slopes. Coiling is more or less planispiral, involute, and slightly skewed in initial volutions. Umbilical depressions are present at both sides and are well developed at the last volution. Spirotheca is three‐layered with a thin tectum and lower and upper tectoria.

*Measurements*: Measurements are given in Tables 1, 2, 3.

*Comparison*: The identified species has a shallow umbilical depression and slightly skewed coiling and resembles the species described by Niko (1987) from the lower part of the Ichinotani Formation of Central Japan (Fukuji discrict, Gifu Prefecture). It can be easily distinguished from other forms of *Eostaffella* in this assemblage. *E*. *igoi* differs from *E*. *parastruvei* Rauser‐Chernousova, 1948a, by having a shallow umbilical depression, slightly skewed coiling, pointed periphery, and smaller proloculus. It is distinguished from *E*. cf. *infulaeformis* Ganelina, 1951, by its lenticular shape with pointed periphery, having slightly skewed coiling in the inner volution, and larger size. *E*. *igoi* differs from *E*. *constricta* by having a larger form ratio and the slightly skewed coiling in the inner volution.

*Occurrence and age*: Latest Viséan–Serpukhovian; Central Japan; Fukuji district; and late Serpukhovian; samples R69; R71; R75; R76; Ghaleh Formation, Asad Abad II section, Sanandaj–Sirjan Zone, Iran.

## 6 MICROFACIES AND PALAEOENVIRONMENT

Based on sedimentary structure, texture, and fossil assemblages, and using the microfacies definition of Dunham (1962), eight major microfacies and two sub‐microfacies were identified in the Asad Abad II section (Figures 12, 13). These microfacies were then categorized along a ramp profile and grouped into four well‐defined depositional environments of the inner ramp that is, the peritidal flat, lagoon, shoal, and open marine. The microfacies types defined in the measured section and corresponding depositional environments are shown in Table 4.

**TABLE 4:** Microfacies types defined in the Asad Abad II section and corresponding depositional environments

Microfacies types (this work)		Microfacies components	Depositional environment	Ramp microfacies types (RMF) (Flügel, 2004)
Crinoidal packstone (MF1)		Crinoid fragments, foraminifers, peloids	Open Marine	RMF7
Bioclastic packstone‐grainstone (MF2)		Bryozoans, foraminifers, crinoid fragments, peloids		RMF14
Coated bioclastic grainstone (MF3)		Coated foraminifers, bryozoans, brachiopods, and crinoids which are accompanied by peloids, intraclasts, and rare ooids	Shoal	RMF26
Bioclastic grainstone (MF4)		Foraminifers, gastropods, crinoids, peloids, algae, and corals		RMF26
Oolitic grainstone (MF5)	Oolitic bioclastic grainstone	Ooids, foraminifers, crinoids, and brachiopods		RMF29
	Sandy oolitic grainstone	Ooids, quartz grains, with rare foraminifers		RMF29
				RMF 29
Mudstone‐wackestone (MF6)		Rare allochems	Lagoon	RMF19
Peloidal grainstone (MF7)		Peloids, sand grains and very fine ooids	Peritidal Flat	‐
Quartz arenite sandstone (MF8)		Quartz grains		‐

### 6.1 Depositional facies

The upper Serpukhovian–lowermost Bashkirian? Ghaleh Formation at the Sanandaj–Sirjan Zone is characterized by well‐defined kinds of textures and skeletal and non‐skeletal grains. The textural types of limestone vary from mudstone, wackestone, and packstone to grainstone. The grains are peloids, ooids, brachiopods, bryozoans, corals, fragmented echinoderms, gastropods, and foraminifers. The microfacies reported here are organized from the open marine to the peritidal environments.

*Crinoidal packstone* (MF1): The main component of this microfacies is crinoid fragments bounded by micrite (Figure 12a). As declared by Scholle and Ulmer‐Scholle (2003), the Palaeozoic crinoids were fully marine, normal salinity organisms and occurred mainly as attached or ‘rooted’ organisms (pelmatozoans). They lived in a variety of shallow, warm water environments, ranging from 3 to 75 m deep. This comparable to RMF 7 defined by Flügel (2004) which indicates the midramp and seaward side of inner ramp depositional environments. MF1 occurs in Unit 2, Sample R3; in Unit 3, Sample R5; and in Unit 4, samples R70 and R71.

*Bioclastic packstone‐grainstone* (MF2): This facies mainly includes an assemblage of bryozoans, and rare crinoids. The matrix consists of micrite to microsparite (Figure 12b). As stated by Scholle and Ulmer‐Scholle (2003), the Palaeozoic bryozoans were more prominent in the tropical to subtropical, shallow water open marine environments with moderate to constant agitation, within the photic zone. The bioclastic packstone‐grainstone can be corresponded with RMF 14 of Flügel (2004). MF2 occurs in Unit 3, samples R17, R18, R22, R24–R26; in Unit 4, samples R58–R60, R64–R66; and in Unit 5, samples R72 and R79.

*Coated bioclastic grainstone* (MF3): This microfacies chiefly includes the coated skeletal grains bounded by sparry calcite cement. The important bioclastic constituents are fusulinoids belonging to Eostaffellidae (Figure 12c). According to Flügel (2004), fusulinoids were adapted to shallow marine, well‐oxygenated, and warm water environments, in depths between a few meters to a few tens of meters. MF3 occurs in Unit 3, samples R4 and R15; and in Unit 4, Sample R57.

Other common bioclasts are brachiopods, crinoids, and gastropods which are accompanied by peloids and rare ooids. This microfacies is the major element of bioclastic shoals or bars. Bioclastic bars form barriers and represent high‐energy shallow water conditions. The coated bioclastic grainstone corresponds to RMF 26 of Flügel (2004).

*Bioclastic grainstone* (MF4): This grain‐supported microfacies is abundant throughout the measured section (Figure 12d,e). It is composed mainly of sparry cement and contains many foraminifers including eostaffellids, endothyrids, and rare archaediscids. The interpretation of shallow water depth is supported by the occurrence of species of Endothyrida and Archaediscida, which live in a variety of shallow water environments. According to summarizing data by Vachard et al. (2010), based on results obtained by Cózar and Rodríguez (2003); Gallagher (1998); and Pille (2008), Endothyrida was endobenthic and/or limited to the water‐bottom interface. As indicated by Vachard et al. (2010), from the Tournaisian to the Serpukhovian, diverse new genera and species of Endothyrida and Archaediscida that developed were limited to the shallow water environments, in temperate to warm‐water and moderate to high energy, well‐oxygenated environments, in depths from several meters to a few tens of meters, more likely of the inner ramp, such as shoals and lagoons, with water of normal salinity. Other common components are crinoids, bryozoans, algae, gastropods, and brachiopods. MF4 occurs in Unit 3, samples R7, R8, R21, R23; in Unit 4, samples R33, R36, R40, R44, R49–R51, R62, R63, and R69; and in Unit 5, samples R73–R76, R80, and R85. The bioclastic grainstone corresponds to RMF 26 and constitutes the high‐energy skeletal shoal sand and banks deposited seaward of ooid shoal, in wave agitated settings.

*Oolitic grainstone* (MF5): This facies is dominated by well‐sorted ooids and skeletal grains, mainly foraminifers. The ooids are up to 0.2 mm and composed of several radial forms, while concentric oolitic grainstones have also been observed. Mud was not observed (Figure 12f). In the report of Flügel (2004), the presence of ooids with sparry calcite suggests very shallow‐water with high‐energy environments of oolitic shoals and currents and not deeper than the outer ramp. This microfacies corresponds to RMF 29 of Flügel (2004). MF5 occurs in Unit 4, samples R34, R37, R38, and R45.

The oolitic grainstone can be classified into two sub‐microfacies, that is, oolitic bioclastic grainstone (Figure 13a) and sandy oolitic grainstone with foraminifers (Figure 13b,c). The oolitic bioclastic grainstone is moderately sorted and exhibits ooid limestone beds rich in foraminifers (mostly eostaffellids and archaediscids) This microfacies occurs in Unit 3, Sample R4.1; and in Unit 4, Sample R39. The other skeletal grains include crinoid fragments, brachiopods, bryozoans, corals, and algae. This facies is almost devoid of terrigenous input.

The latter sub‐microfacies, that is, the sandy oolitic grainstone, is characterized by moderate to well‐sorted ooids and the quartz grains. This microfacies mainly occurs under high‐energy, shallow water environments in platform margin of sand shoals (Cózar et al., 2006; Flügel, 2004). The sandy oolitic grainstone facies occurs in Unit 3, Sample R4; and in Unit 5, samples R77, R81, and R84. This microfacies is equivalent to RMF 29 of Flügel (2004).

*Mudstone‐wackestone* (MF6): Due to high abundance of micrite and scarcity of allochems this microfacies is named as the wackestone‐mudstone microfacies (Figure 13d). Mud‐supported texture and scarce fauna characterize a restricted lagoonal setting (Colombié & Strasser, 2005). This microfacies is comparable with RMF 19 of Flügel (2004). MF6 occurs in Unit 2, Sample R2; in Unit 3, samples R6, and R32; and in Unit 4, Sample R67.

*Peloidal grainstone* (MF7): The peloidal grainstone mainly consists of peloids in sparitic cement. Peloids are small, well sorted, and irregularly shaped. This microfacies is generally barren of fossils. Other allochems are rare sand grains and very fine ooids (Figure 13e). MF7 occurs in Unit 3, samples R27–R30; in Unit 4, samples R31, R41–R43, R46, R47, R56, and R61.

*Quartz arenite sandstone* (MF8): The quartz arenitic sandstone contains more than 90% rounded to subrounded quartz grains (Figure 13f) which are bounded by fine‐grained sparry calcite. It shows a major fall in sea level. MF8 occurs in Unit 3, samples R9–R14, R16, R19, R20; in Unit 4, samples R35, R48, R52–R55, R68; and in Unit 5, samples R78, R82, and R83.

### 6.2 Palaeoenvironmental model

As noted above, the carbonate succession of the Ghaleh Formation at the Asad Abad II section is proposed to have been deposited along a ramp profile and is grouped into four well‐defined depositional environments of the inner ramp, that is, the peritidal flat, lagoon, shoal, and open marine.

The occurrence of crinoidal packstone (MF1, Figure 12a) and bioclastic packstone‐grainstone with bryozoans (MF2, Figure 12b) at the base of this interval indicates deposition under the wave activity. Wave activity is evidenced by the presence of crinoidal packstone that was mainly deposited in the open‐marine environments or basin‐ward of the oolitic shoal, at or around the wave base, with moderately agitated conditions (Al‐Tawil & Read, 2003; Della Porta et al., 2005).

The shoal facies are defined by the significant occurrence of coated bioclastic grainstone with foraminifers (MF3, Figure 12c); bioclastic grainstone (MF4, Figure 12d,e), oolitic grainstone (MF5, Figure 12f), oolitic bioclastic grainstone with archaediscid foraminifers (MF5, Figure 13a), and sandy oolitic grainstone (MF5, Figure 13b,c). These facies were essentially deposited in a high‐energy environment, above the wave base or in the areas of constant wave action (e.g., Al‐Tawil & Read, 2003; Cózar et al., 2006; Della Porta et al., 2005; Flügel, 2004).

The significant occurrence of micrite and scarcity of allochems (MF6, Figure 13d) indicates the low‐energy of the lagoonal facies which occur in association with shallow‐marine deposits (e.g., Al‐Tawil & Read, 2003; Barnaby & Ward, 2007).

The appearance of peloidal grainstone (MF7, Figure 13e) and quartz arenite sandstone (MF8, Figure 13f) indicates the peritidal facies. As declared by Al‐Tawil and Read (2003), the peloidal grainstone is interpreted as the coastal aeolinates which are devoid of any in‐situ fossils and have plentiful peloid grains and very fine to fine sands. Joachimski (1994) described this microfacies as a tidal channel facies. According to Chamley et al. (1997), besides, a similar microfacies is described in a channel and shallow pool environment. Colombié and Strasser (2005) considered the tidal channels in a back barrier setting or tidal flat environment.

Based on summarizing data by Atakul‐Özdemir et al. (2011) that come from results obtained by Barnaby and Ward (2007) and Fischer and Sarnthein (1988), the quartz arenite sandstone is generally deposited during the regressive phase and records sea‐level lowstands.

## 7 CONCLUSIONS

The interesting and rather well‐preserved assemblages of foraminiferal faunas of the upper Serpukhovian–lowermost Bashkirian? is reported for the first time from the Asad‐Abad II section of the Ghaleh Formation in the Sanandaj–Sirjan Zone, Iran.

The successive occurrences of taxa in the section make it possible to distinguish four assemblages of foraminifers: namely, (1) an assemblage with *B*. *minima* and *E*. *paraprotvae*, (2) an assemblage with *B*. *cribrostomata*, (3) an assemblage with *P*. *utkaensa* and *Plectostaffella* spp., and (4) an assemblage with *Plectostaffella* ex gr. *varvariensis*. The assemblages contain some species that are used as markers for the upper Serpukhovian and lower Bashkirian deposits in different regions, which made it possible to identify the foraminiferal assemblages. *E*. *paraprotvae*, *Eostaffella* ex gr. *postmosquensis*, *Plectostaffella acuminulata*, *P*. *utkaensa*, *Plectostaffella* ex gr. *varvariensis*, *B*. *cribrostomata*, *B*. *concinna*, *B*. *minima*, and *Globivalvulina moderata* are among the characteristic taxa. The greatest taxonomic diversity is shown by the assemblage with *P*. *utkaensa* and *Plectostaffella* spp. which makes it possible to correlate the deposits with the top of the Serpukhovian.

Based on sedimentary structure, texture, and fossil assemblages, eight major microfacies and two sub‐microfacies were identified which suggest the moderate to high‐energy shallow marine warm environments, more likely of the inner ramp.

## AUTHOR CONTRIBUTIONS

Shirin Fassihi collecting and analysis the data, writing the paper, preparying the figures. Elana Kulagina analysis tha data, revising and writing the biostratigraphic part of the paper. Refining the analyzed data. Preparying the plates. Petra Heinz refining the analyzed data on the palaeoenvironment and microfacies. Analysis and checking the depositional facies part of the manuscript. Fariba Shirezadeh preparying part of material and the logistic support.

## CONFLICT OF INTEREST

The authors declare that they have no known competing financial interests or personal relationships that could have appeared to influence the work reported in this paper.

### PEER REVIEW

The peer review history for this article is available at https://publons.com/publon/10.1002/gj.4641.

## Data Availability

Data sharing not applicable to this article as no datasets were generated or analysed during the current study.
